# Thyroid hormone signaling in ocular development and diseases

**DOI:** 10.1186/s40659-025-00618-1

**Published:** 2025-07-02

**Authors:** Pengbo Zhang, Yan Nie, Nan-Ji Lu, Qianfeng Jiao, Xiaofang Wang, Xibo Zhang, Longqian Liu

**Affiliations:** 1 Department of Ophthalmology, West China Hospital, Sichuan University, Chengdu, China; 2 Laboratory of Optometry and Vision Sciences, West China Hospital, Sichuan University, Chengdu, China; 3 West China School of Public Health and West China Fourth Hospital, Sichuan University, Chengdu, Sichuan China; 4 State Key Laboratory of Southwestern Chinese Medicine Resources, School of Pharmacy, Chengdu University of Traditional Chinese Medicine, Chengdu, China; 5 Department of Ophthalmology, Affiliated Hospital of Southwest Medical University, Luzhou, Sichuan China

**Keywords:** Thyroid hormone signaling, Ocular development, Ocular diseases, Deiodinases

## Abstract

Thyroid hormone (TH) signaling plays a pivotal role in maintaining metabolic homeostasis across multiple organ systems, including the eye. Proper ocular development depends on precise regulation of TH levels, as deviations from this dynamic range can result in ophthalmopathy. Emerging evidence underscores the involvement of TH signaling in various ocular pathologies, such as diabetic retinopathy. Notably, suppressing TH signaling has been shown to preserve cone photoreceptors in mouse models of retinal degeneration, highlighting the intricate interplay between TH signaling and photoreceptor viability. Additionally, the well-documented association between abnormal thyroid function and proptosis further emphasizes the critical role of TH signaling in orbital tissue homeostasis. The biological effects of TH are mediated through its binding to thyroid hormone receptors (TRs), which initiate downstream genomic and non-genomic pathways. Of particular interest is the role of deiodinases (DIOs), which modulate local TH signaling in a tissue- and temporally specific manner, independent of systemic TH levels. Despite the recognized importance of TH signaling in ophthalmopathy, significant gaps remain in our understanding of its cellular and molecular mechanisms. Future studies focusing on TH signaling within specific ocular cell lineages are essential for elucidating the underlying mechanisms and uncovering new therapeutic opportunities. This review aims to provide a comprehensive overview of TH signaling in ocular physiology and pathology, with an emphasis on advancing our understanding of its molecular mechanisms.

**Graphical abstract:**

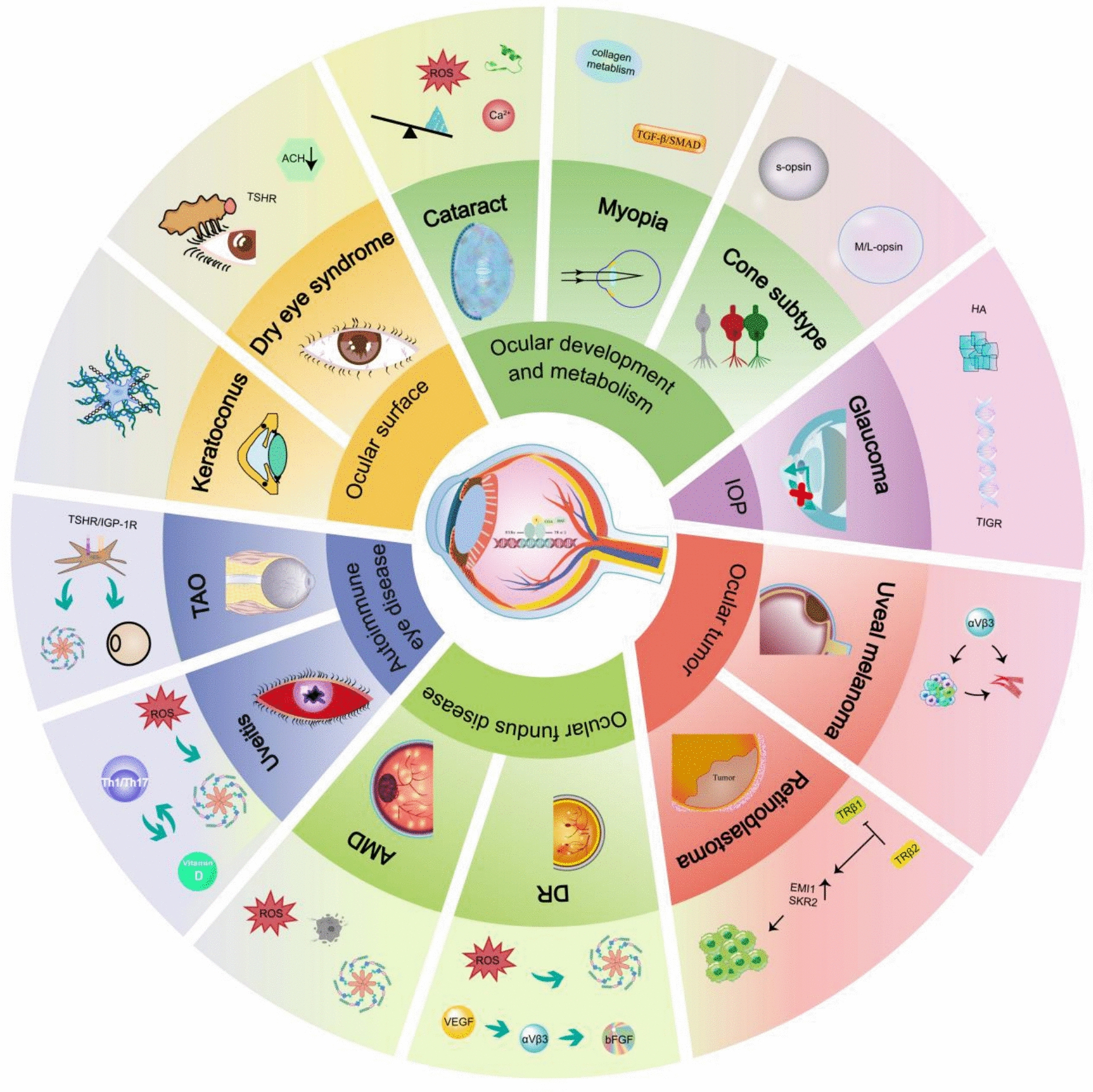

## Introduction

Thyroid hormone (TH) is essential for cellular differentiation, systemic development and aging, as well as metabolic regulation in almost all tissues and cells. The dynamic balance of TH is fundamental to organ function, and its imbalance is linked to the onset of various diseases, including cardiovascular, hepatic, neurodegenerative, and ocular disorders [1–4]. From a clinical perspective, both hypothyroidism and hyperthyroidism are closely associated with ocular diseases such as thyroid-associated ophthalmopathy (TAO), diabetic retinopathy (DR), and age-related macular degeneration (AMD) [4]. 

TH signaling has been shown to be crucial for retinal development and function, determining the fates of cone subtypes [5]. Disruption of this signaling pathway can impair cone differentiation, which may contribute to color vision defects. For instance, preterm infants (i. e., infants born before full term) with low TH levels exhibit a higher likelihood of developing such defects, likely due to impaired cone development [6–9]. Single-cell analyses further reveal that Müller glia coordinate retinal intercellular communication via TH signaling during light/dark adaptation [10]. This highlights the nuanced role of TH signaling in both normal visual physiology and adaptive responses.

Despite these vital effects of TH signaling in the visual system, its physiological and pathological roles remain incompletely understood. Increasing evidence indicates that disruption in local and systemic TH signaling regulation contributes to various ocular pathologies. This review summarizes current knowledge of the effects of TH signaling on ocular development and ophthalmopathy, highlighting its influence throughout the lifecycle of living organisms. Given the central role of TH signaling in these processes, targeted modulation of this pathway offers significant potential for halting ocular disease progression and guiding the development of novel therapeutic agents. 

### Global and local TH signaling

**Fig. 1:**
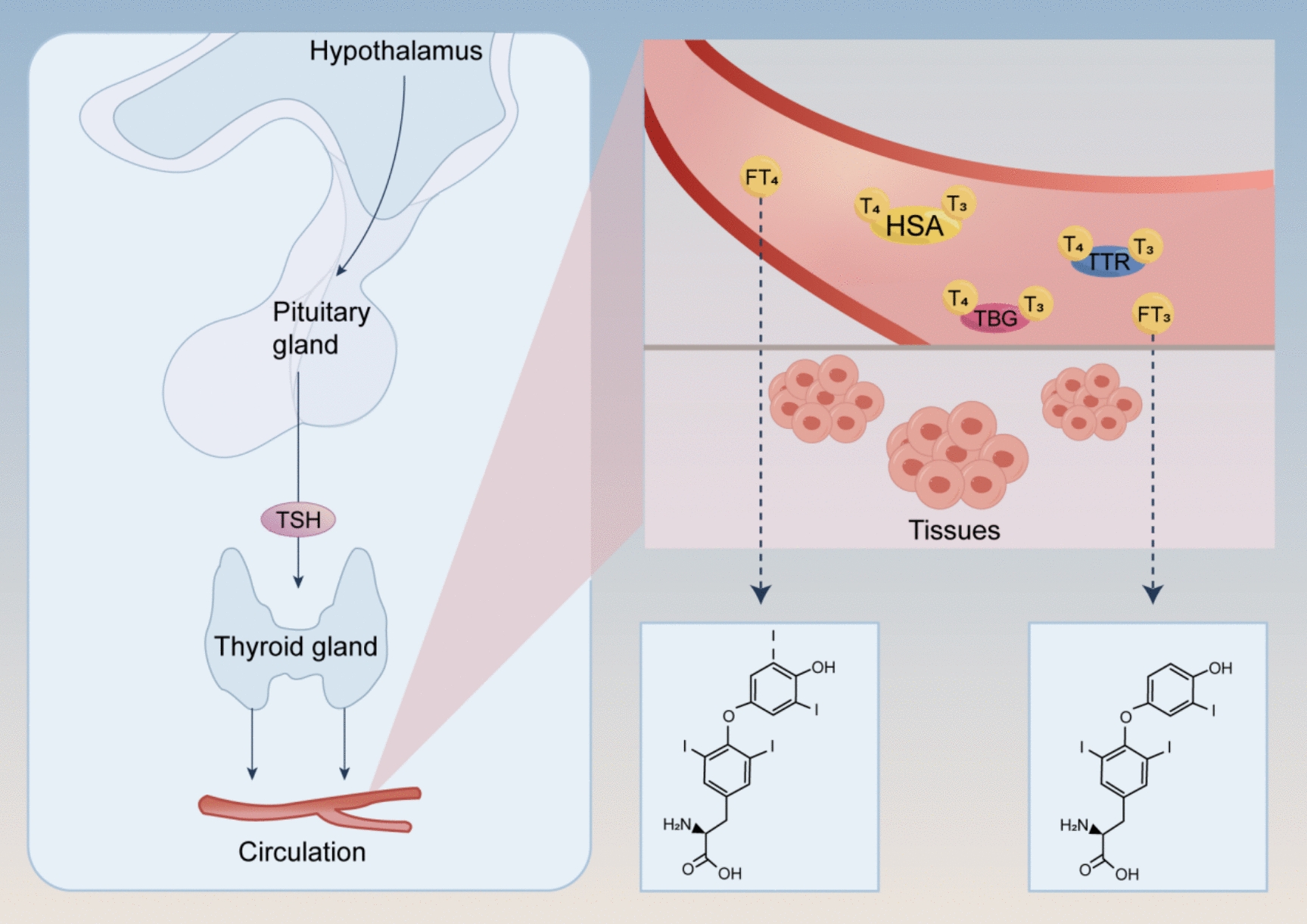
TH (T3 and T4) are synthesized in the thyroid gland from tyrosine and iodide, regulated by the HPT axis. The hypothalamus releases TRH, stimulating the pituitary to release TSH, which then prompts the thyroid to produce T3 and T4. High levels of T3 and T4 inhibit TRH and TSH secretion, maintaining balance. While T4 is more abundant, T3 is more biologically active. Most circulating TH is bound to carrier proteins like TBG, TTR, and HSA, with only FT3 and FT4 being active and able to enter cells

TH, including triiodothyronine (T3) and thyroxine (T4), is synthesized in the thyroid gland from tyrosine and iodide. Their production and release into circulation are tightly regulated by the hypothalamus-pituitary-thyroid (HPT) axis, a finely tuned feedback system [11]. The HPT axis involves the hypothalamus secreting thyrotropin-releasing hormone (TRH), the pituitary gland releasing thyroid-stimulating hormone (TSH), and the thyroid gland synthesizing and releasing TH. Importantly, circulating T3 and T4 levels can inhibit TRH and TSH secretion, maintaining homeostasis throughout the lifecycle [12]. Among circulating TH, T4 is the predominant form, but T3 has greater receptor-binding activity. Most circulating TH is bound to carrier proteins, including T4-binding globulin (TBG), transthyretin (TTR), and human serum albumin (HSA), which act as reservoirs. Only the free fractions of T3 and T4 (FT3 and FT4) are biologically active and capable of crossing cell membranes to exert physiological effects [13] (Fig. 1).

**Fig. 2:**
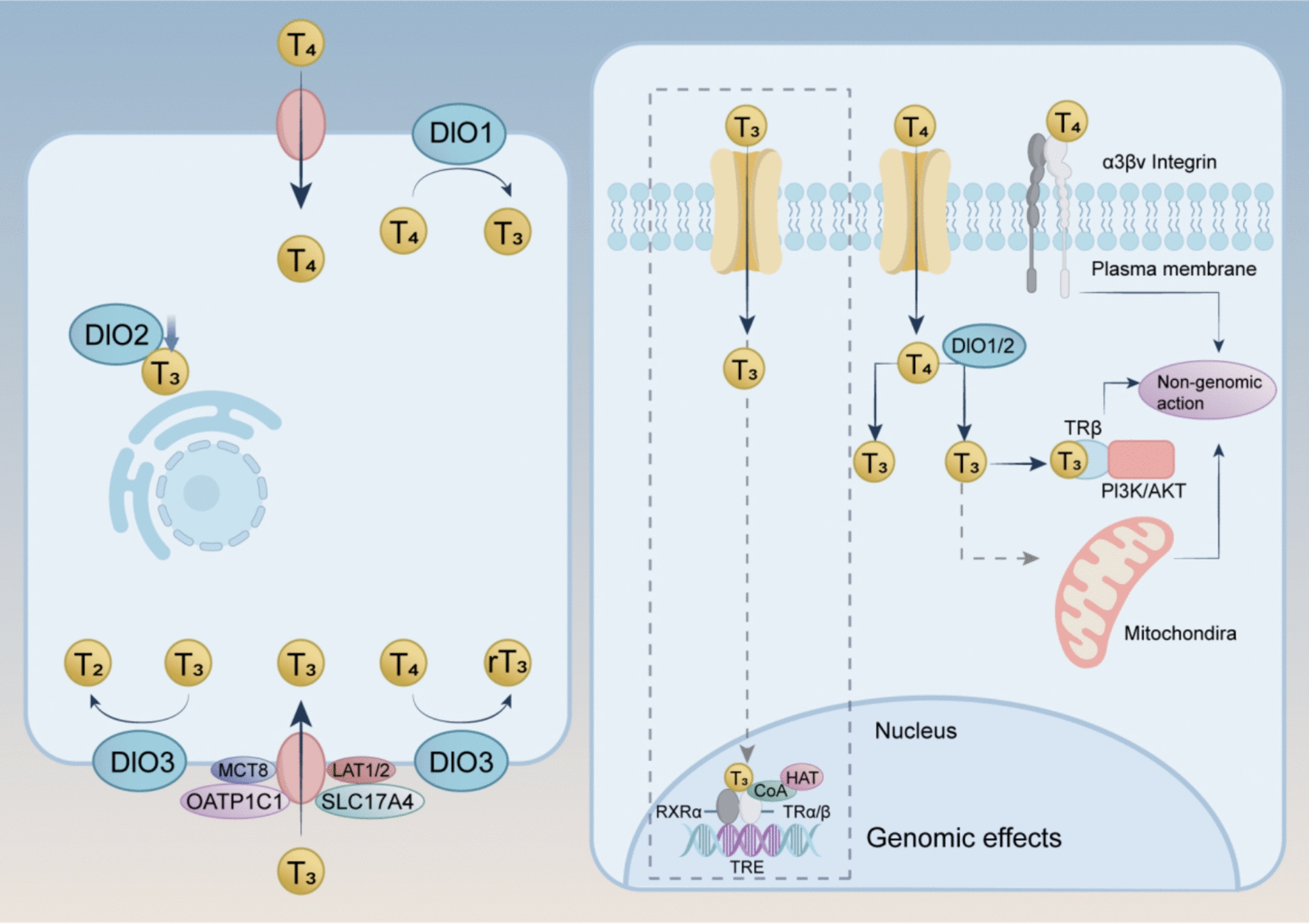
TH is transported into cells by specific transporters like OATP1C1, MCT8, SLC17A4, and LAT1/2. Inside cells, the activity of DIOs (DIO1, DIO2, and DIO3) regulates TH levels. DIO1 and DIO2 convert T4 to the active form, T3, while DIO3 inactivates T4 and T3 by converting them to rT3 and T2. This local regulation allows precise control of TH levels independent of the systemic levels. T3-bound TRs interact with specific DNA sequences, known as TREs, either as homodimers or as heterodimers with retinoid X receptors (RXRs). Upon T3 binding, THRs recruit nuclear coactivators (CoAs), which, in turn, are associated with histone acetyltransferases (HATs), facilitating gene transcription. The transcriptional targets of TRs include primary responsive genes that contain TREs within their promoter or enhancer regions. Additionally, non-genomic effects involve the interaction of T3 or T4 with cellular proteins such as PI3K/AKT and the αvβ3 integrin receptor, as well as mediating mitochondrial-related effects

At the cellular level, specific transporter proteins facilitate TH uptake. These include organic anion-transporting polypeptide (OATP)1C1, monocarboxylate transporter (MCT)8, SLC17A4, and L-type amino acid transporters (LAT1 and LAT2), which mediate cell-specific TH transport [14, 15]. Local regulation of TH signaling depends on the activity of three iodothyronine deiodinases (DIOs; DIO1, DIO2, and DIO3). These enzymes can fine-tune intracellular TH concentrations independently of systemic levels [16–18]. DIO2 is primarily localized in the endoplasmic reticulum, while DIO1 and DIO3 are situated in the plasma membrane [19]. DIO1, predominantly expressed in the liver, kidneys, and thyroid, and DIO2, mainly found in the central nervous system, pituitary, skeletal muscle, and brown adipose tissue, catalyze the conversion of T4 into the active form, T3 [20, 21]. Conversely, DIO3, primarily located in the brain, placenta, and pancreas, inactivates T4 and T3 by converting T4 to reverse T3 (rT3) and T3 to diiodothyronine (T2) [20, 21] (Fig. 2) (Table. 1).

**Table 1:** Comparative Functional and Spatial Characteristics of Deiodinase Isoforms in Systemic and Local Thyroid Hormone Regulation

Deiodinase	Primary function	Serum T3 regulation	Serum T4 regulation	Local T3 regulation	Local T4 regulation	Systemic vs local action	Localization	Refs
DIO1	Converting T4 to T3 and rT3	Minor T3 contributor;Activity increases in hyperthyroidism	Facilitating T4 clearance; Activity increases in hyperthyroidism	Local T3 production in liver, thyroid and kidney; Rapid equilibrium between intra/extracellular T3	Deiodinating T4 locally, stabilizing systemic T4 levels	Systemic; mainly circulating T3/T4 levels but minor role in local regulation	Plasma membrane; Liver, thyroid and kidney	[20, 360, 361]
DIO2	Converting T4 to T3;	Major T3 contributor; Activity increases in hypothyroidism	Modulating T4 to T3 conversion; Activity decreases in hyperthyroidism	Fine-tunes T3 availability in specific tissues (brain, muscle, brown adipose) independently of serum T3	Regulating intracellular T4 metabolism; Being ubiquitinated to control T3 production	Primarily local; contributes significantly to serum T3, especially in hypothyroidism	Endoplasmic reticulum; CNS, pituitary, skeletal muscle, and brown adipose tissue	[20, 360–363]
DIO3	Converting T4 and T3 to rT3 and T2; main pathway for T3 clearance	Primary contributor to plasma T3 clearance; Activity increases in response to elevated serum T3 levels	Enhancing T4 clearance, particularly in pathological conditions	Reduces local T3 levels, particularly in placenta, CNS and hemangiomas	Converting T4 to rT3; preventing T3 activation in specific tissues	Primarily local; reduces T3/T4 availability, with indirect effects on serum levels in normal and disease states	Plasma membrane; Brain, placenta, and pancreas	[364, 365]

T3, as the major biologically active form, exerts its function through both genomic and non-genomic mechanisms [22]. Thyroid hormone receptors (TRs), belonging to the nuclear hormone receptor superfamily, mediate these effects [23]. TRs exist in two main isoforms, TRα and TRβ. In their genomic actions (transcriptional), T3-bound TRs bind to thyroid hormone response elements (TREs) on chromatin, initiating gene transcription through dynamic interactions with co-repressor and co-activator complexes. In the absence of T3, TRs associate with co-repressors, suppressing transcription. Upon T3 binding, co-repressors are released, and co-activators are recruited, shifting transcriptional repression to activation [24–26]. Non-genomic actions involve T3 or T4 interacting with cytoplasmic proteins, such as PI3K/AKT or TR-associated partners, and initiating rapid signaling cascades. In parallel, T3 can also stimulate mitochondrial respiration through direct intracellular mechanisms. These rapid, transcription-independent pathways may ultimately influence gene expression indirectly [27, 28].

Under normal physiological conditions, local TH levels are primarily regulated by the balance between tissue and serum T3 concentrations, mediated by DIOs [29, 30]. The effects of DIOs are integrated. Serum TH levels directly influence the activity of DIOs: an increase in serum T3 leads to reduced DIO2 activity and enhanced DIO3 activity, while a decrease in serum T3 results in the opposite effect. These adjustments allow tissues to finely regulate local T3 concentrations in accordance with systemic changes, ensuring alignment between tissue-specific TH demands and overall homeostasis. Moreover, DIO1 in peripheral tissues, such as the liver and kidney, plays a key role in converting T4 to T3, contributing to the replenishment of serum T3 levels and reinforcing systemic TH homeostasis [20] (Fig. 2) (Table 1).

TH signaling is tightly controlled in both space and time, governed by the expression patterns of DIOs. However, in pathological conditions, this regulatory balance can be disrupted. Altered activity of DIOs, driven by factors such as inflammation, hypoxia, or dysregulated signaling pathways, can result in tissue-specific TH signaling that is uncoupled from serum TH levels. This local decoupling of TH activation allows tissues to respond autonomously to microenvironmental stressors, potentially contributing to disease progression [16]. The expression of DIOs is modulated by a range of endogenous signals, including sonic hedgehog, nuclear factor-κB (NF-κB), growth factors, bile acids, and hypoxia-inducible factor-1α (HIF-1α). Exogenous factors further expand DIOs functionality, influencing juvenile development, metabolic regulation, tissue repair, injury response, and energy homeostasis [16, 31–35]. In the retina and other ocular tissues, dysregulated DIOs expression may alter key processes such as angiogenesis and oxidative stress adaptation, underscoring the importance of DIOs-mediated local TH signaling in both physiological and pathological ocular conditions.

### TH developmental actions on the eye

TH plays an essential role in ocular development, beginning in the embryonic stage and extending into early postnatal life. During the chicken embryonic period, maternally deposited TH is utilized by the embryo until embryonic day 6 (E6). By E9, the embryonic thyroid gland initiates endogenous synthesis of T4 and T3, resulting in a substantial rise in plasma and ocular TH levels by E10 [36]**.** Experimental studies suggest that TH is essential for determining the overall size and morphology of the eyeball and its adnexal structures, including the orbit, eyelids, and nasolacrimal system [37–41]**.** In a rat model of congenital hypothyroidism, hypothyroid embryos exhibit smaller size of eyeball and significantly reduce the optic primordia area and volumn compared to controls, along with delayed eye closure. Postnatally, hypothyroid rats show delayed eye opening accompanied by persistent structural deficits, including thinning of the photoreceptor and ganglion cell layers, impaired photoreceptor outer segment morphogenesis [41]. Consistent with these findings, clinical studies have reported that children with congenital hypothyroidism may exhibit impairments in visual processing, including deficits in color vision, visual-motor integration, and visual cognition [7, 42, 43]**.** However, neonatal screening studies report no significant differences in ocular biometry or motility between children with congenital hypothyroidism and healthy controls, likely due to early and effective hormonal therapy [37].

### TH signaling in retinal development and maturation

**Fig. 3:**
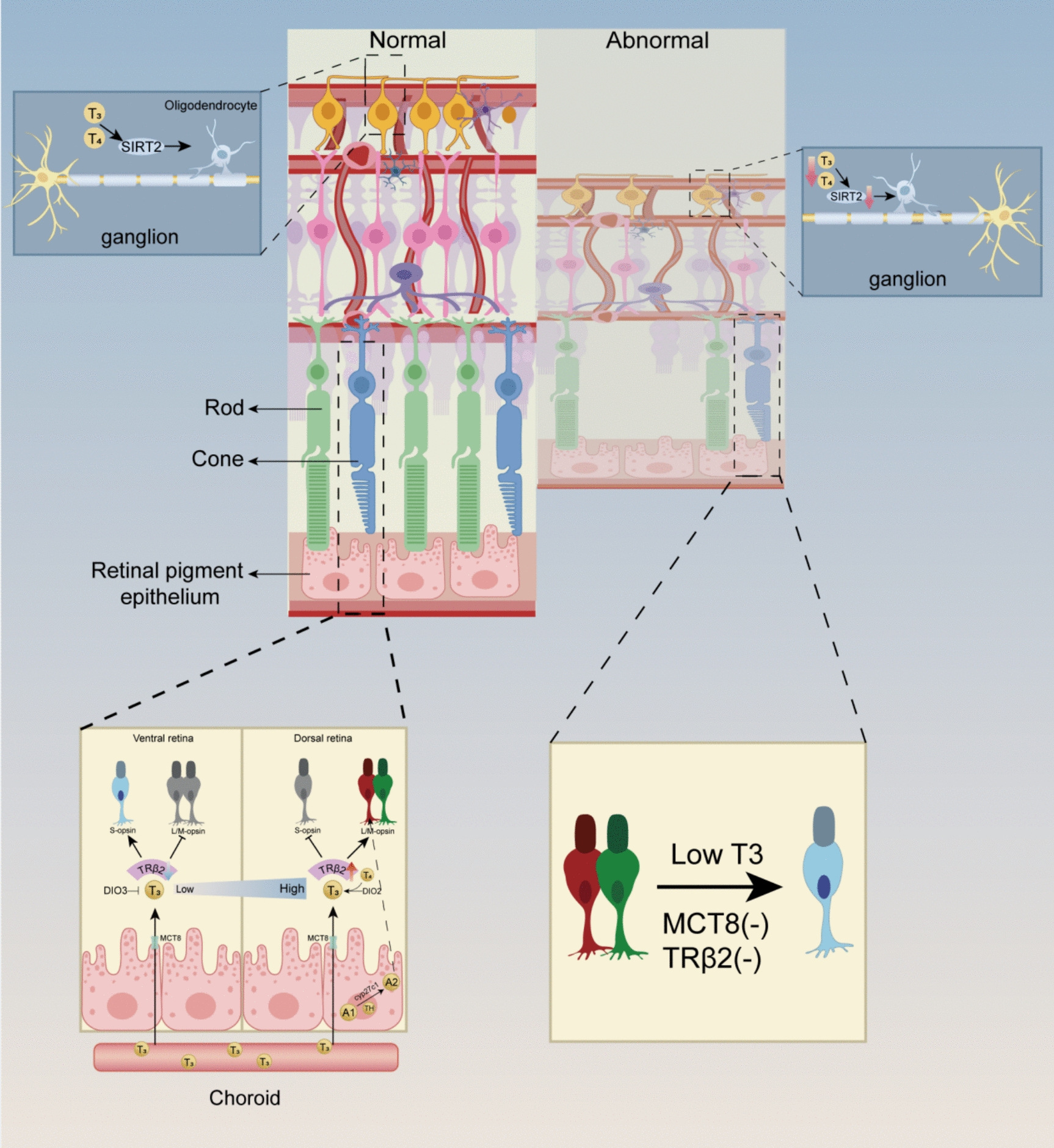
TH signaling is essential for retinal development, regulating cone photoreceptor fate, retinal layer formation, optic nerve myelination, and RPE maturation. T3 directs cone opsin specification, with early S-opsin expression transitioning to M/L-opsin in a TH-dependent manner, guided by TRβ2. DIOs (DIO2 and DIO3) fine-tune local T3 availability, ensuring proper timing of photoreceptor differentiation. TH also promotes oligodendrocyte differentiation and optic nerve myelination by regulating SIRT2 expression. In the RPE, TH transport via MCT8 and enzymatic regulation of cyp27c1 impact photoreceptor function and spectral tuning. These mechanisms highlight the crucial role of TH in coordinating retinal maturation at multiple levels

*TH-driven cone photoreceptor specification* TH signaling plays a fundamental role in retinal development and maturation, as demonstrated across multiple species, including amphibians [44], pisces [45], rodents [46, 47], birds [48], and human-derived retinal organoids [5]. The retina, as part of the central nervous system, exhibits high sensitivity to TH levels, with its differentiation and structural organization critically dependent on T3-mediated transcriptional regulation [46]. Among its essential functions, TH governs cone photoreceptor cell fate determination, retinal layer formation, optic nerve myelination, and the functional maturation of the retinal pigment epithelium (RPE) (Fig. 3).

The human retina comprises three types of cone opsins: S-opsin (short-wavelength-sensitive), M-opsin (medium-wavelength-sensitive), and L-opsin (long-wavelength-sensitive). Studies using human retinal organoids have revealed that TH plays a pivotal role in cone subtype specification, where S-opsin cones develop first, followed by L/M-opsin cones in a TH-dependent manner. This transition is orchestrated by nuclear TRs, which serve as key transcriptional regulators guiding photoreceptor maturation [5].

In the developing mouse retina, S- and M-opsins exhibit a dorsoventral gradient with opposing patterns of expression. During fetal development until birth, T3 is uniformly distributed at low concentrations, promoting default S-opsin expression. However, from postnatal day 0 (P0) onward, T3 levels increase asymmetrically, with higher concentrations in the dorsal retina and lower concentrations in the ventral retina by P10. This region-specific T3 distribution directs M-opsin expression dorsally while maintaining S-opsin expression ventrally [40]. Consistent with these findings, hypothyroid mouse and amphibian models display delayed photoreceptor maturation, increased S-opsin levels, and reduced M-opsin expression [46, 49–51], whereas exogenous T3 accelerates cone differentiation, favoring M/L-opsin specification in both embryonic rat and fetal human retinal cells [52]. In zebrafish larvae, T3 exposure modulates cone opsin expression, promoting M/L-opsin at the expense of S-opsin, mirroring findings from human retinal organoids [53].

*TH regulation of retinal architecture and optic nerve maturation* Beyond photoreceptor differentiation, TH signaling shapes retinal cytoarchitecture. In hypothyroid rats, retinal thickness is significantly reduced, with disruptions particularly evident in the photoreceptor and ganglion cell layers [41]. Additionally, TH promotes optic nerve myelination by driving oligodendrocyte differentiation, a process crucial for retinal ganglion cell (RGC) axonal maturation [54–56]. Oligodendrocyte precursor cells in the optic nerve begin myelination during early postnatal development, and T3 has been confirmed to directly induce oligodendrocyte differentiation [56]. Notably, Sirtuin 2 (SIRT2), a key modulator of neuroprotection and differentiation, is positively influenced by TH signaling. Recent studies suggest that TH deficiency leads to reduced SIRT2 expression in the ganglion cell and visual cell layers, indicating that TH may upregulate SIRT2 activity, potentially through thyroid receptor (TR) pathways. This regulation of SIRT2 could contribute to myelination and neuronal differentiation, supporting retinal cytoarchitecture maturation. Collectively, these findings underscore the broader role of TH in shaping retinal neurons and their glial support networks, particularly in processes such as axonal maturation and myelination, as demonstrated in preclinical models [57].

*Spatiotemporal regulation of T3 availability by DIOs* Precise spatiotemporal control of T3 levels is crucial for retinal development, with DIOs orchestrating localized T3 bioavailability. In early retinal organoid development, high DIO3 activity restricts T3 signaling, preserving progenitor cell proliferation and S-opsin specification. As maturation progresses, DIO2 becomes dominant in the dorsal retina, elevating regional T3 levels and inducing M/L-opsin differentiation [5]. In neonatal mice, *DIO3* gene loss-of-function mutations results in premature T3 elevation, leading to cone-selective apoptosis and impaired visual responses, highlighting the necessity of tight temporal regulation of T3 levels [58].

In zebrafish, DIO3b expression peaks early in eye development, maintaining low intracellular T3 concentrations [59]. *DIO3* knockdown disrupts cone differentiation and visually guided behavior, further confirming its critical role in retinal structural formation and functional maturation [39]. Importantly, *DIO2* and *DIO3* exhibit dynamic, region-specific expression, emphasizing a layered regulatory mechanism that governs retinal TH availability at distinct developmental stages. Further studies should aim to map precise temporal shifts in the activity of DIOs across different retinal compartments.

*TRβ2 and TRβ1: isoform-specific roles in retinal development* In addition to TH availability, TR isoform dynamics critically shape cone differentiation. TRβ2, a cone-specific TH receptor, exhibits biphasic expression [47, 60–62], with an initial peak coinciding with cone precursor generation and a secondary peak during the S- to M/L-opsin transition [40, 60]. Knockdown of *TRβ2* in zebrafish and rodents reduces M/L-opsin expression and increases S-opsin levels, reinforcing its role as a lineage determinant for M/L cones [40, 47, 50, 53, 63, 64].

TRβ2 directly drives cone subtype differentiation, whereas TRβ1 plays a distinct regulatory role in retinal maturation. TRβ1 expression peaks later in development, and although it is detected in cones, *TRβ1*-knockout mice exhibit only minor changes in opsin expression and retain normal electroretinographic responses, suggesting a limited role in direct photoreceptor specification [65]. Instead, TRβ1 may influence postnatal retinal remodeling, synaptic connectivity, or metabolic homeostasis, warranting further investigation.

### TH influence on RPE function

TH signaling also plays a crucial role in RPE function and its interactions with photoreceptors. The RPE serves as a gateway for TH transport, as evidenced by studies on MCT8, a membrane TH transporter. In mouse models, loss of *MCT8* gene in the RPE induces hypothyroid-like changes, including a shift from M- to S-cone specification, which can be partially reversed by reintroduction of *MCT8* gene [66].

TH also regulates the transcription of RPE enzymes. A key example is cytochrome P450 family 27 subfamily C member 1 (CYP27C1), which catalyzes the conversion of vitamin A1 (the opsin chromophore) to vitamin A2 [53, 63, 67, 68]. This conversion reduces red-shifted spectral sensitivity, thereby diminishing the capacity to detect near-infrared wavelengths, as observed in experimental models. Given that TH induces *cyp27c1* gene expression in zebrafish, this mechanism likely represents a TH-mediated shift favoring L-cone spectral tuning [53, 63]. Furthermore, TH modulates RPE structural integrity, with T3 inhibition via propylthiouracil (PTU) reducing RPE layer thickness, reinforcing its role in RPE morphogenesis and function [69].

#### TH signaling in corneal and lens development

TH plays a pivotal role in corneal transparency, nerve development, and lens homeostasis, acting through both genomic and non-genomic mechanisms to regulate cellular differentiation, extracellular matrix (ECM) composition, and metabolic processes. Through TR-mediated transcriptional control, TH modulates key structural and enzymatic proteins essential for corneal clarity and lens function. Disruptions in TH signaling can lead to corneal thickening, impaired nerve innervation, and increased susceptibility to oxidative stress-induced cataract formation, underscoring the necessity of TH homeostasis in ocular development.

*TH regulation of corneal transparency and endothelial function* The corneal endothelium and epithelium are essential for maintaining corneal transparency by regulating hydration and ionic balance. In chick embryos, T4 enhances corneal dehydration and transparency by promoting endothelial interdigitation, ensuring optimal stromal architecture for light transmission [70–73]. Conversely, thiouracil, a thyroid peroxidase (TPO) inhibitor that blocks the iodination of tyrosine residues in thyroglobulin (Tg) and subsequently reduces the biosynthesis of T3 and T4, disrupts these processes, leading to corneal thickening, delayed epithelial maturation, and impaired transparency [72, 73].

At the molecular level, TH regulates corneal transparency through modulation of keratan sulfate proteoglycan (KSPG)-related genes and carbonic anhydrase genes, both of which are crucial for ECM organization and fluid homeostasis [74, 75]. Alterations in KSPG concentration and sulfation patterns regulated by TH affect corneal hydration and refractive stability [74]. Additionally, TH enhances ion transport mechanisms, ensuring precise hydration control and maintaining optical clarity [74, 75]. The interplay between TH signaling and corneal ECM remodeling suggests that impaired TH homeostasis may predispose to corneal opacities and stromal dystrophies.

*TH regulation of corneal nerve development* Corneal nerve development is tightly regulated by spatiotemporal signaling cues, with TH playing a key role in directing trigeminal nerve patterning and maturation. During early embryonic development, trigeminal axons initially avoid the corneal periphery, forming a pericorneal nerve ring before extending radially into the corneal stroma [71, 75]. T3 accelerates the formation of this nerve ring, independent of overall embryonic growth rate, by enhancing the responsiveness of corneal nerves to guidance cues [71, 75].

Following the establishment of the nerve ring, both T3 and T4 stimulate corneal nerve growth and branching, ensuring proper innervation of the corneal stroma and epithelium [75]. This suggests that TH signaling may modulate the activity of neurotrophic factors or axon guidance molecules, facilitating corneal sensory function. Given that corneal nerves are crucial for maintaining epithelial homeostasis and wound healing, disruptions in TH signaling may impair corneal sensitivity, epithelial repair, and overall ocular surface integrity. Collectively, these observations support a key role for TH in corneal neurodevelopment, with potential implications for neurotrophic keratopathy and other corneal neuropathies.

*TH regulation of lens structure and cataractogenesis* The lens, like the cornea, is highly dependent on TH signaling for structural integrity and metabolic regulation. Among thyroid hormones, T4 has been shown to exert protective effects against oxidative stress-induced cataract formation, primarily by modulating lipid metabolism in lens epithelial cells.

One of the key mechanisms underlying TH-mediated lens protection involves T4-induced reductions in lipid peroxidation and serum glucose levels, as demonstrated in developing chick embryos [76]. By altering the composition and turnover of membrane lipids, T4 helps maintain lens transparency and prevents oxidative damage [77, 78]. Rather than activating classical antioxidant pathways, TH protects the lens primarily by remodeling mitochondrial membrane lipids. It increases cardiolipin content (critical for inner mitochondrial membrane structure and cytochrome c anchoring), induces mitochondrial membrane hyperpolarization (elevated ΔΨm), and reduces lipid unsaturation, thereby lowering oxidative sensitivity. These changes collectively diminish lipid peroxidation and prevent lens protein aggregation, contributing to cataract prevention [77, 78]. The interplay between TH signaling, lipid homeostasis, and oxidative stress response suggests that TH may function as an endogenous protective factor against cataractogenesis.

## TH signaling and ocular surface pathology

TH signaling plays a multifaceted role in maintaining ocular surface integrity by regulating corneal structure, tear production, and glandular homeostasis. Clinical and experimental studies have revealed significant correlations between thyroid dysfunction and alterations in corneal morphology and biomechanics.

### Corneal morphology and keratoconus

Multiple clinical investigations have demonstrated that patients with hyperthyroidism often exhibit thinner corneas and abnormal tomographic parameters, features commonly associated with the development of keratoconus [79]. Conversely, hypothyroidism is associated with a reversible increase in corneal thickness [80]. The observed association between thyroid dysfunction and keratoconus has been consistently documented across multiple clinical investigations [81–85], suggesting that TH signaling may play a regulatory role in maintaining corneal structural integrity. However, conflicting data exist—some reports indicate thicker corneas in hyperthyroid patients compared to euthyroid controls [86]. These discrepancies likely reflect variations in age, sex, ethnicity, and systemic TH levels, underscoring the complexity of TH-related corneal effects.

### Mechanisms of corneal remodeling

In cultured monkey corneas, exogenous T4 administration has been shown to upregulate T4 receptor (T4R) expression and directly modulate collagen synthesis and extracellular matrix composition in a dose-dependent manner, leading to altered collagen profiles within the stromal compartment [83]. Similar results were observed in ex vivo corneal tissues from keratoconus patients, where T4 exposure induced collagen and cytokeratin-13 expression in keratocytes, but not in epithelial layers [83]. Additionally, a recent in vitro study showed that 1.0 µg/ml thyroxine significantly increased Collagen I protein expression in keratoconus-derived human corneal fibroblasts (KC-HCFs), but not in keratoconus keratocytes [87]. Thyroxine also enhanced cell proliferation in both normal and KC-derived keratocytes, without significantly altering TGF-β1 or Collagen V expression [87]. These findings suggest that T4 may selectively influence collagen production and cell behavior in a context- and cell type-dependent manner.

Elevated FT4 concentrations in the aqueous humor [84] and tear fluid [88] of keratoconus patients suggest localized TH dysregulation at the ocular surface. One hypothesis posits that the lacrimal gland may synthesize and secrete T4 into the tear film, thereby influencing corneal curvature and biomechanical stability—though this remains speculative and warrants further investigation [88].

### Dry eye disease and meibomian gland dysfunction

In addition to its effects on corneal morphology, TH signaling plays a pivotal role in the pathogenesis of dry eye disease (DED), particularly in the context of thyroid-associated ophthalmopathy (TAO) and autoimmune thyroid disorders [89]. Patients with active TAO often exhibit severe meibomian gland dysfunction (MGD), with substantial structural loss of the glands contributing to tear film instability and exacerbating DED severity [90–93]. Even among patients with inactive TAO, a higher rate of meibomian gland dropout is observed compared to non-TAO controls [94]. Furthermore, patients with Graves’ disease (GD)—even in the absence of TAO-related exophthalmos—demonstrate impaired tear film homeostasis and corneal abnormalities [95], reinforcing the broader impact of thyroid dysfunction on ocular surface health.

### TH effects on lacrimal gland physiology

Mechanistic studies have identified several TH-regulated processes in the lacrimal gland. In chronic hypothyroidism, TRβ expression is upregulated in lacrimal tissue, resulting in decreased acetylcholine release from parasympathetic neurons and reduced tear secretion [96]. Animal models support this link: in hypothyroid horses, facial nerve dysfunction leads to keratoconjunctivitis sicca, which is reversible with levothyroxine therapy [97]. In rats, thyroidectomy reduces lacrimal gland size, while T4 supplementation promotes polyploid cell formation and glandular hypertrophy, independent of growth hormone or testosterone influence [98]. Similar effects have been documented in rabbits, where T4 stimulates tear production and supports lacrimal gland regeneration [99].

### Autoimmune pathways involving TSHR

A key autoimmune mechanism involves the aberrant expression of thyroid-stimulating hormone receptor (TSHR) in the lacrimal gland. As TSHR is a primary autoantigen in TAO, its local expression may render the lacrimal gland susceptible to immune-mediated damage, thereby compounding the effects of MGD and further destabilizing the tear film in thyroid-related conditions [100].

**Fig. 4:**
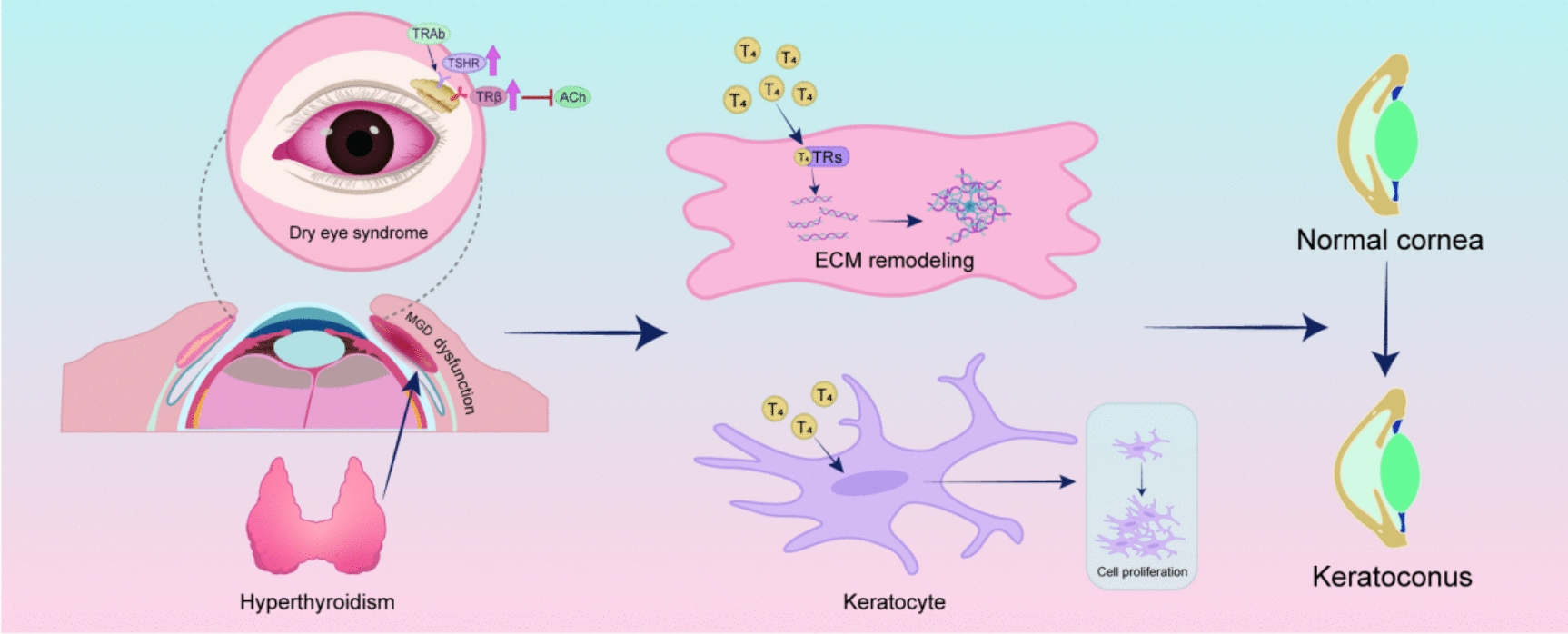
Thyroid hormone signaling regulates ECM remodeling and ocular surface homeostasis.Ex vivo studies using cultured monkey corneas demonstrate that T4 modulates collagen expression and ECM composition in a dose-dependent manner, accompanied by upregulation of T4R in stromal keratocytes. In vitro, T4 promotes collagen I synthesis in keratoconus-derived corneal fibroblasts and enhances proliferation in both normal and keratoconus keratocytes, indicating a cell-type-specific response. Elevated tear free T4 levels in keratoconus may reflect local dysregulation rather than systemic excess. In the lacrimal gland, TRβ overexpression under chronic hyperthyroid conditions suppresses acetylcholine release and tear secretion, while aberrant expression of TSHR may exacerbate autoimmune-mediated glandular dysfunction

Taken together, these findings highlight TH signaling as a central regulator of ocular surface physiology, influencing both corneal biomechanics and lacrimal gland function. Dysregulation of TH signaling—whether due to hyperthyroidism, hypothyroidism, or localized TH imbalance—can contribute to the onset and progression of ocular surface disorders, including keratoconus and DED. Future studies should aim to clarify the molecular pathways by which TH signaling modulates ECM remodeling, lacrimal gland function, and tear film dynamics, with the ultimate goal of identifying new therapeutic targets for TH-related ocular surface disorders (Fig. 4).

## TH signaling and glaucoma

Glaucoma encompasses a spectrum of progressive optic neuropathies characterized by optic nerve damage and visual field loss, with elevated intraocular pressure (IOP) serving as a major risk factor. Emerging observational and experimental studies suggest a potential role for TH in the pathophysiology of glaucoma. Numerous studies have shown that both clinical and subclinical hypothyroidism are associated with elevated IOP and an increased risk of glaucoma development [80, 101–106]. A clinical observation suggest that hypothyroid patients undergoing TH replacement therapy may experience improved IOP control and enhanced aqueous outflow, implying a possible role for TH balance in ocular pressure regulation[107]. However, contradictory reports exist, with some studies finding no significant association between hypothyroidism and glaucoma [108–110]. These inconsistencies may arise from confounding factors, such as co-morbidities like hypertension and diabetes [111, 112], which are independently linked to glaucoma risk [113, 114] and may contribute to an overestimation of the hypothyroidism-glaucoma relationship if not properly accounted for [108].

Clinical reports have suggested that patients with TAO may have an increased predisposition to elevated IOP and glaucoma, with this risk potentially correlating with TAO severity [115–121]. Despite the link between TAO and glaucoma, little attention has been given to the potential interrelation between hyperthyroidism (without TAO) and glaucoma. A Danish population-based cohort study found no significant increase in glaucoma risk among hyperthyroid patients without TAO [122], suggesting that systemic hyperthyroidism alone is not a major factor. However, another observational study suggested that although hypothyroidism itself is not directly associated with open-angle glaucoma (OAG), thyroid treatments, particularly current T4 use (OR 2. 1; 95% CI 1. 0–4. 4) or a history of thyroid surgery (OR 2. 5; 95% CI 1. 0–6. 2), may independently contribute to OAG [123].

Mechanistically, hypothyroidism may promote IOP elevation through its effects on the trabecular meshwork, a key structure in the regulation of aqueous humor outflow. In hypothyroid states, reduced T3 levels have been associated with the accumulation of hyaluronic acid (HA) within the trabecular meshwork, leading to increased resistance to aqueous humor outflow and subsequent IOP elevation [124–126]. Importantly, this process is reversible with the restoration of normal TH levels, highlighting the dynamic nature of TH regulation in trabecular meshwork function [124–126]. Moreover, T3 has been implicated in modulating the expression of the trabecular meshwork-inducible glucocorticoid response (*TIGR*) gene, also known as myocilin, which plays a key role in regulating outflow resistance [127]. T3 may counterbalance mechanical or stress-induced upregulation of *TIGR* expression, potentially protecting against excessive outflow obstruction [127].

In TAO, the mechanisms underlying IOP elevation differ from those in hypothyroidism. The primary factor is mechanical compression resulting from increased intraorbital tissue volume, including inflamed extraocular muscles and orbital fat, which can physically impede aqueous humor outflow [119, 128]. Contraction of the extraocular muscles against intraorbital adhesions exacerbates this effect, further elevating IOP [119, 128]. Additionally, autoimmune and endocrine factors, including excessive TH or TSH receptor autoantibodies, may contribute to disease progression through local inflammatory or fibrotic processes within the trabecular meshwork [124, 129]. Although significant strides have been made in elucidating the role of TH signaling in glaucoma, the precise molecular pathways remain to be fully characterized, warranting further investigation.

## TH signaling and cataract

Cataract, the leading cause of vision impairment and blindness globally, is characterized by the progressive opacification of the lens [130–132]**.** Age-related cataract (ARC), also known as senile cataract, is the most common form of cataract. Although hypothyroidism is not a classic risk factor for cataract development [133], several epidemiological studies have suggested a potential association [134, 135]. For example, adult patients with a history of congenital hypothyroidism have been reported to exhibit an increased incidence of cataracts in some retrospective studies [134]. Mendelian randomization (MR) studies have also provided genetic evidence that both hypothyroidism [135] and hyperthyroidism [136] may contribute to an increased risk of ARC. However, not all studies are consistent; one investigation reported no significant association between hyperthyroidism and ARC, suggesting that the relationship may depend on additional factors, such as disease duration and severity [135]**.**

The potential for TH supplementation to influence metabolic cataracts has been postulated in select reports, though no definitive conclusions can be drawn at this time [137]. While TH supplementation may alleviate specific metabolic disturbances, excessive TH exposure has been associated with moderate cortical opacities in some observational studies, suggesting a potential dose-related risk [138]. Cataract formation has also been observed following thyroidectomy, potentially linked to altered calcium-phosphorus metabolism due to incidental parathyroid gland removal [139]. However, cases of thyroidectomy-associated cataracts have been reported even in the absence of parathyroid involvement, with blue-dot cataracts being the most common form observed, characterized by punctate opacities within the lens [140]. Several mechanisms have been proposed to explain the role of TH signaling in cataractogenesis: Lens Protein Dysregulation: TH deficiency may impair the synthesis and turnover of lens structural proteins, such as crystallins, leading to the accumulation of misfolded or damaged proteins, which promotes lens opacification [135]; Water Metabolism Disruption: TH plays a crucial role in maintaining cellular and tissue water balance. Hypothyroidism-induced water retention within the lens may contribute to osmotic stress, fiber swelling, and eventual cataract formation [141]; Oxidative Stress: Oxidative stress is a well-established factor in cataract development. Hypothyroidism has been linked to increased oxidative damage in the lens, not may by impaired antioxidant enzyme activity, but through alterations in mitochondrial membrane lipid composition—such as reduced cardiolipin content and increased lipid unsaturation—that elevate oxidative susceptibility and promote lipid peroxidation [77, 78, 142]; Calcium Dysregulation: TH deficiency can indirectly affect calcium homeostasis, leading to calcium accumulation within the lens, a known trigger for cataract formation [143–146].

These mechanisms provide a multifaceted understanding of how imbalances in TH signaling can influence lens transparency. Although hypothyroidism and hyperthyroidism appear to contribute to cataract formation through overlapping and distinct mechanisms, further studies are needed to establish causality and assess whether targeted modulation of TH signaling could serve as a therapeutic approach to prevent or delay cataract development.

## TH signaling and retina disease

### TH signaling and DR

DR is a severe vision-threatening complication of diabetes mellitus (DM), primarily resulting from chronic hyperglycemic damage to retinal microvasculature, which is classified as non-proliferative DR (non-PDR) or proliferative DR (PDR) [147–149]. The interplay between thyroid dysfunction and DR is gaining recognition due to the established relationship between thyroid disease and DM. Several studies have documented a higher prevalence of thyroid disorders among patients with DM and vice versa [150, 151].

Subclinical hypothyroidism, a mild thyroid dysfunction characterized by mildly elevated TSH and normal circulation free TH levels, is associated with an increased risk of DR and its severity in patients with type 2 diabetes mellitus (T2DM) [152–154]. Elevated TSH levels have been independently associated with retinal arteriolar narrowing and reduced arteriovenous ratios, suggesting their role in microvascular pathology [155]. Even within the normal range, higher TSH levels have been linked to an increased risk of DR in well-controlled T2DM patients [156]. Conversely, higher FT3 and FT4 levels are negatively correlated with retinal microangiopathy, indicating the protective role of adequate TH levels in maintaining retinal microvascular health [157–159]. Impaired central and peripheral TH sensitivity further exacerbates DR progression, highlighting the complex role of TH signaling in retinal pathology [160]. Retrospective clinical data suggest that T4 replacement therapy may be associated with a lower risk of DR in hypothyroid T2DM patients compared to those without treatment [161].

In type 1 diabetes mellitus (T1DM), the relationship between DR and autoimmune thyroid disease is particularly strong [162]. Children with co-occurring T1DM and autoimmune thyroiditis exhibit worsened retinal parameters, suggesting that thyroid autoimmunity exacerbates retinal dysfunction [163]. A recent MR study suggested a genetic predisposition linking autoimmune thyroid disease to increased DR risk in T1DM and lower T2DM susceptibility, highlighting the distinct pathophysiological roles of thyroid dysfunction across diabetes subtypes [164].

However, contrasting evidence exists in adult T1DM patients, where thyroid autoimmunity is negatively correlated with retinal microangiopathy, suggesting a potential protective effect of autoimmune thyroid disease in this population [165]. This unexpected finding may be explained by immune-modulatory mechanisms that reduce vascular inflammation and damage. Supporting this, higher FT3 levels are associated with a lower prevalence of retinal microvascular alterations in euthyroid adult T1DM patients, further underscoring the protective role of optimal TH signaling [166]. In a Brazilian cohort, lower TSH levels (0. 4–2. 5 mU/L) were statistically associated with a lower risk of DR and renal failure in T1DM patients, independent of glycemic control and disease duration [167]. In a small case series (n=19), some T1DM patients showed apparent improvements in retinal hemorrhages and exudates following TH therapy (12 out of 19 cases); however, these observations are anecdotal and not supported by statistical significance [168].

**Fig. 5:**
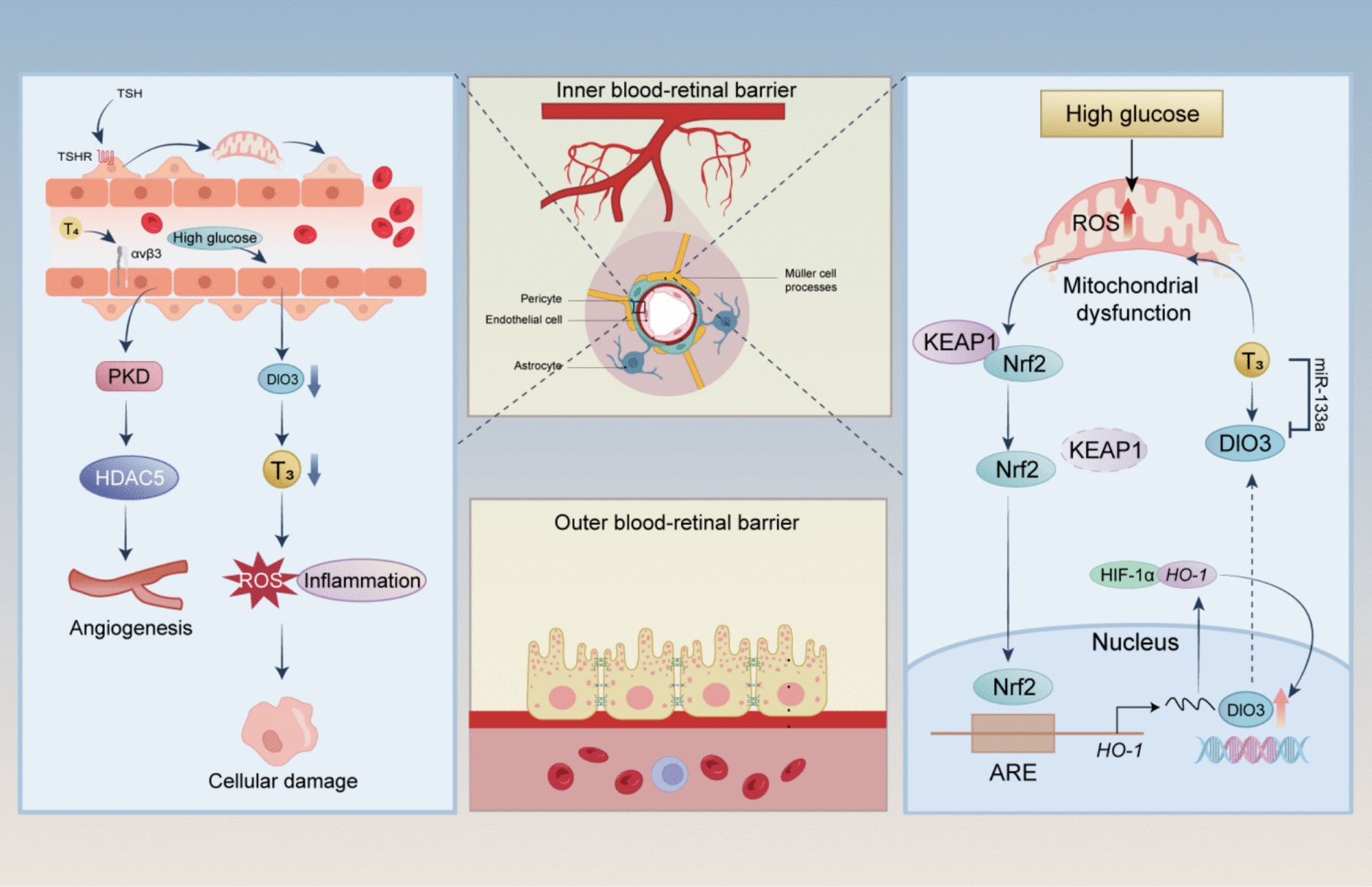
TH signaling contributes to DR through multiple pathways. Elevated TSH induces retinal pericyte apoptosis via mitochondrial dysfunction, while TH promotes endothelial migration via the integrin αvβ3/PKD/HDAC5 pathway. In retinal endothelial cells, high glucose downregulates DIO2, reducing T3 levels, exacerbating oxidative stress, inflammation, and endothelial cell death. In MIO-M1 cells, high glucose-induced oxidative stress activates Nrf2/HO-1/HIF-1, upregulating DIO3 and sustaining a low T3 state. This suppresses TR activation, downregulates miR-133a, and worsens mitochondrial dysfunction, driving DR progression

Mechanistically, the impact of TH signaling on DR pathogenesis is primarily mediated through the following pathways (Fig. 5). Vascular regulation: The HPT axis is critical to retinal development and vascular homeostasis [169]. In vitro studies have identified functional TSHR on human retinal pericytes [170] and experimental elevation of TSH has been shown to exacerbate high glucose-induced pericyte apoptosis through mitochondrial dysfunction, contributing to DR progression [170]. In diabetic rats, T4 and T3 levels were progressively reduced over time, leading to impaired vascular reactivity [171] —a condition reversible with T4 supplementation [172]; Inflammation and VEGF Pathways: Chronic inflammation and VEGF-driven angiogenesis play key roles in DR progression [173]. Elevated serum VEGF levels are observed in patients with untreated Graves’ disease (GD) and Hashimoto’s thyroiditis (HT), which positively correlated with increased vascularization [174]. Subclinical hypothyroidism is associated with higher serum C-reactive protein (CRP) levels, suggesting that thyroid dysfunction exacerbates DR through pro-inflammatory pathways [175]. This is further supported by studies showing elevated levels of IL-6 and TNF-α in subclinical hypothyroidism patients, which are positively correlated with endothelial dysfunction and systemic inflammation [176]. In patients with DR, key subunits of the integrin β2 subtype—including the integrin β subunit CD18 and the α subunits CD11a and CD11b—are significantly upregulated, indicating that integrins play a role in the pathogenesis of DR [177]. Additionally, TH, through the integrin αvβ3/PKD/HDAC5 pathway, upregulates bFGF to promote endothelial cell migration, proliferation, and angiogenesis, while high glucose and hypoxia induce VEGF and erythropoietin (EPO) expression, activating the integrin αvβ3/ERK1/2 pathway to drive pathological angiogenesis, and targeting integrin αvβ3 with Tetrac presents a potential therapeutic strategy for DR [151, 178, 179]; Oxidative stress: thyroid dysfunction can contribute to insulin resistance [180], dyslipidemia [181, 182], and oxidative stress [183, 184], collectively promoting vascular damage. Some evidence suggests that the diabetic retina may experience a localized reduction in intracellular T3 levels [185]. Similar reductions have been observed in other metabolically active tissues-such as the heart, liver, and skeletal muscle-where they may occur either in the context of normal circulating thyroid hormone concentrations, due to tissue-specific alterations in deiodinase activity, or as part of a systemic non-thyroidal illness syndrome (NTIS), in which both serum and tissue T3 levels are reduced despite preserved thyroid gland function [186–188]. Supporting this, a recent study demonstrated that high glucose conditions reduce DIO2 expression in retinal tissue, leading to decreased T4-to-T3 conversion and an increased T4/T3 ratio, ultimately lowering intracellular T3 levels [189]. Functionally, this T3 deficiency has been shown to exacerbate oxidative stress-likely involving nitric oxide-derived species such as peroxynitrite (ONOO⁻), as well as mitochondrial superoxide (O₂⁻)-and to upregulate inflammatory markers in retinal endothelial cells (RECs), thereby promoting chronic inflammation, endothelial cell death, and the progression of diabetic retinopathy [189]. In parallel, in MIO-M1 cells, high glucose-induced oxidative stress activates Nrf2/HO-1/HIF-1 signaling, upregulating DIO3 and promoting a self-sustaining low T3 state loop, where reduced T3 levels suppress TR activation, downregulate miR-133a, further enhancing DIO3 expression, and ultimately lead to mitochondrial dysfunction and metabolic failure in DR [185]. Future research should focus on the regulation and targeted intervention of local TH signaling in the retina to provide new perspectives for the treatment of DR.

### TH signaling and AMD

AMD is a progressive retinal disorder affecting the macula, where critical neural, structural and vascular layers undergo degenerative changes, ultimately leading to cell death [190], which is classified into two categories: dry (non-neovascular) and wet (neovascular). AMD is a primary cause of serious vision loss in the elderly and is anticipated to affect nearly 288 million individuals worldwide by 2040 [190]. Clinically, thyroid dysfunction has been studied for its association with a heightened risk of AMD development. An earlier observational study identified a link between AMD and hypothyroidism in adults aged 50 and above [191], however, subsequent findings revealed that overt hyperthyroidism in older populations is also independently associated with an increased risk of AMD [192]. Recent epidemiological data suggest that individuals with a history of hyperthyroidism or hypothyroidism may face significantly elevated risks—up to eightfold and threefold, respectively—of developing AMD in later life [193]. Supporting this, a nationwide longitudinal cohort study in Denmark reported that both hypo- and hyperthyroidism were statistically associated with increased risk of exudative AMD [194].

Biochemical analyses have revealed that patients with wet AMD exhibit significantly elevated serum FT4 levels, while total T3 and TSH concentrations remain relatively stable [195]. Unlike DR, where thyroid function shows different trends, higher FT4 levels in euthyroid individuals have been positively correlated with both an increased risk of AMD and RPE alterations, particularly in populations aged 55 and older [196]. Furthermore, a two-sample MR study suggested that genetic variants associated with higher normal-range FT4 levels are linked to an elevated AMD risk [197]. A follow-up bidirectional two-sample MR analysis clarified that this risk is primarily evident in the late stages of AMD progression [164]. Additionally, geographic atrophy, a severe form of dry AMD, has been associated with higher rates of TH use [198, 199] in retrospective analyses, although some studies report no significant correlation between thyroid medication and AMD risk despite a clear association between thyroid disorders and AMD [200, 201].

Mechanistic investigations into TH signaling provide insights into the pathogenesis of AMD. Studies have shown that inhibition of TH signaling or deficiency of TRs exerts protective effects against oxidative stress-induced cell death in RPE and photoreceptors in an AMD mouse model [202, 203], may primarily by attenuating TH-driven oxidative injury pathways including DNA damage (γH2AX, 8-OHdG), lipid peroxidation (NOX4/GPX4 axis), and ROS-dependent activation of necroptotic signaling cascades (e.g., RIPK1/RIPK3/MLKL), as well as proinflammatory gene expression (e.g., TNF-α, IL-6) [202, 203]. Notably, excessive TH signaling has been implicated in photoreceptor degeneration, possibly through mechanisms involving oxidative stress, necroptosis, and inflammation [204]. Specific receptor subtypes play distinct roles in retinal pathology: the deletion of TRα1 and TRβ provides protection to RPE cells, rods, and cones, while deletion of TRβ2 specifically protects RPE cells and cones but not rods [203]. These findings emphasize the need for further exploration of the differential roles of TR subtypes in retinal diseases.

Therapeutic strategies targeting the regulation of TH components in the retina are emerging as promising approaches. For instance, suppressing TH signaling has been shown to enhance photoreceptor survival across multiple retinal degeneration models [205]. In retinal degeneration mouse models, subretinal delivery of AAV5-IRBP/GNAT2-DIO3 (DIO3 expression specifically in cones) increased cone density by 30–40% [206]. Additionally, intravitreal and topical administration of the DIO2 inhibitor iopanoic acid significantly improved cone survival compared to untreated controls [206]. Targeting DIOs and intracellular regulation of TH components locally in the retina may represent a novel strategy for retinal degeneration diseases including AMD. These findings suggest that local modulation of TH-regulating enzymes, such as DIO2 and DIO3, may represent a novel therapeutic avenue for managing retinal degenerative diseases, including AMD. Further research should aim to elucidate the precise molecular pathways by which TH signaling influences retinal cell fate and identify optimal strategies for clinical translation.

## TH signaling and myopia

Myopia, also referred to as near-sightedness or short-sightedness, is a prevalent vision condition that typically arises during childhood or early adulthood. It occurs when excessive axial elongation of the eye causes incoming light from distant objects to focus in front of the retina, resulting in blurred vision for distant objects [207]. Myopia is one of the most widespread ocular conditions globally, affecting 10–30% of the adult population in many regions and up to 80–90% of young adults in parts of East and Southeast Asia [208–210]. This rapid escalation of myopia, particularly in younger populations, underscores its growing public health significance [208–210].

Emerging evidence suggests a potential link between thyroid function and myopia. A clinical study examining thyroid function and ocular parameters revealed that lens power is significantly increased in patients with clinical hypothyroidism and shows a negative correlation with FT4 levels [86]. In children and adolescents, a reduction in lens power has been observed both before and during the onset of myopia, implying that altered lens dynamics may serve as an early indicator of myopia development [211, 212]. In a clinical study involving euthyroid individuals, spherical power (SP) was found to be higher in those with positive thyroid autoantibodies compared to antibody-negative counterparts [86]. SP also showed a positive correlation with levels of anti-Tg antibody, anti-thyroid peroxidase antibody, and Tg, while a negative correlation was observed between SP and TSH levels [86], highlighting the complexity of thyroid-autoimmune interactions in refractive error development.

Clinical observations in TAO have raised the possibility that TH signaling may contribute to refractive changes. A case report including 5 patients with progressive TAO reported that orbital soft-tissue expansion exerts mechanical effects on the globe, leading to posterior globe flattening and hyperopic shifts [213]. This hyperopic shift can be reversed by orbital decompression, which restores globe contour [213]. Notably, patients undergoing orbital decompression often experience significant myopic shifts due to axial elongation [214]. These changes predominantly involve alterations in axial length (AL), spherical refraction, and spherical equivalent (SE), while anterior chamber depth and lens thickness remain largely unaffected [214]. Moreover, the changes in AL and SE are significantly correlated, suggesting that TAO mainly causes refractive changes by affecting AL of the eye [214]. Supporting this, thyroid pathology (primarily hyperthyroidism) and exophthalmos (≥17 mm) are associated with poorer visual acuity and higher prevalence of myopic refraction [215]. Additionally, by assessing at different ages, myopia is also found to be more common among teenagers and adults with Graves'disease [216]. These preliminary clinical findings raise the hypothesis that TH signaling may be involved in myopic axial elongation.

The myopic shift observed in TAO or after decompression surgery raises the possibility that local TH signaling within ocular tissues plays a significant role in myopia development. This hypothesis warrants further investigation, particularly focusing on how local TH signaling influences axial elongation and scleral remodeling.

A study of metabolomics has explored the pathogenic mechanism of pathological myopia at the molecular and metabolic levels. The metabolic pathway of TH synthesis is significantly enriched in both vitreous humour and aqueous humor of patients with pathological myopia [217]. Serum analyses further demonstrate that TTR levels, a key TH transport protein, are significantly elevated in patients with high myopia and correlate with increased axial length and decreased visual acuity [218]. Elevated vitreous TTR concentrations have also been detected in patients with high myopia complicated by macular detachment or macular holes [219]. In cases of macular detachment, TTR exhibits an abnormally stable tetrameric structure, potentially resulting from protein misfolding, which may exacerbate retinal damage [219]. Collectively, these findings suggest that dysregulated TH transport contributes to the pathological elongation of the eye and associated complications. TTR may serve as both a biomarker and a pathogenic factor in myopia progression, offering potential targets for future therapeutic interventions.

TH signaling is intricately linked to collagen metabolism and ECM remodeling, both of which are central to myopia pathogenesis. Collagen degradation and abnormal ECM remodeling in the sclera contribute to the biomechanical weakening and elongation of the eye [220, 221]. Mechanistic studies have shown that TH, particularly T3, can regulate collagen synthesis, metabolism and fibroblast function [222]. T3 binds to TRs and antagonizes the transcriptional activity of the TGF-β/SMAD pathway by reducing SMAD-binding element occupancy and histone acetylation, thereby suppressing collagen transcription [223]. This antagonistic effect on TGF-β signaling may be relevant to myopia, as TGF-β is a key regulator of scleral fibroblast transdifferentiation and ECM remodeling during axial elongation. Notably, TH signaling has been shown to inhibit lung fibrosis in mice by improving epithelial mitochondrial function [224] and enhancing alveolar cell differentiation through Kruppel-Like Factor 2 (KLF2) and CCAAT-enhancer binding protein alpha (CEBPA) [225]. Although these studies are primarily focused on pulmonary tissues, they highlight the broader regulatory role of TH signaling in tissue remodeling, which could extend to the sclera. Investigating whether similar pathways are involved in scleral fibroblast function and collagen metabolism is a promising avenue for future research.

## TH signaling and autoimmune eye disease

### TH signaling and uveitis

Uveitis refers to inflammation of the uveal tissues of the eye, including the iris, ciliary body, and choroid. Uveitis can be classified based on anatomical location and infectious etiology. Anatomically, it is categorized into anterior, intermediate, posterior, and panuveitis, depending on the primary site of inflammation [226]. Based on etiology, uveitis is classified as either infectious or non-infectious, with non-infectious uveitis occurring in association with systemic autoimmune diseases or autoimmune condition localized to the eye [227]. Uveitis is a major cause of vision loss, with more than one-third of affected individuals experiencing visual impairment [228]. In developed countries, it ranks as the fifth or sixth leading cause of blindness, contributing to approximately 10–15% of all blindness cases [229, 230].

In 1915, researchers first proposed the use of thyroid extract to treat malignant uveitis, suggesting a potential link between thyroid function and uveitis [231]. Several epidemiological studies have reported an association between thyroid dysfunction and uveitis onset. Thyroid disease and uveitis may share common autoimmune mechanisms, as a population-based case-control study found that individuals with thyroid disease had a 1. 7 to 1. 8-fold increased risk of uveitis compared to controls, even after adjusting for age, sex, race, smoking status, and autoimmune disease history [232]. Similarly, a nationwide cohort study in Taiwan found an increased incidence of uveitis among individuals with thyroid disorders, particularly in those without metabolic comorbidities such as diabetes or hypertension, strengthening the thyroid-uveitis association supports a specific autoimmune-mediated link rather than a general inflammatory process or systemic comorbidities [233]. Supporting this, a reduction in serum T4 levels has been observed in certain uveitis patients [234], and 2. 3% of individuals with uveitis have been diagnosed with GD [235]. Furthermore, patients with uveitis often exhibit impaired peripheral utilization of TH [236]. However, some studies have found no definitive link between thyroid dysfunction and non-infectious uveitis [237].

Uveitis is a heterogeneous inflammatory disease that can occur as a primary ocular disorder or as a manifestation of systemic autoimmune conditions, reflecting complex underlying immune mechanisms. Thyroid dysfunction may be closely associated with specific subtypes of uveitis. For instance, Tubulointerstitial Nephritis and Uveitis (TINU) syndrome has been primarily linked to hyperthyroidism [238–240], though a case report the association between TINU and Hashimoto's thyroiditis, which is the most common cause of hypothyroidism [241]. Additionally, Vogt-Koyanagi-Harada (VKH) syndrome has been associated with both hyperthyroidism and hypothyroidism [242–248], indicating that thyroid signaling may influence the uveal tissues through systemic immune regulation. Notably, uveitis related to Human T-Cell Leukemia Virus Type 1 (HTLV-1) shows a specific correlation with GD and hyperthyroidism, reinforcing the concept of immune-mediated thyroid-ocular interactions [249–256]. Behcet’s disease (BD) is closely associated with the development of uveitis. Studies have found that approximately 35% of BD patients have positive serum thyroglobulin antibodies (TGA) [257]. Additionally, some reports indicate that although thyroid function remains normal in BD patients, their pituitary response to TRH stimulation is reduced, suggesting potential involvement of the HPT axis [258].

From a mechanistic perspective, thyroid dysfunction may influence uveitis through shared autoimmune mechanisms, oxidative stress, endocrine-immune crosstalk, and vitamin D-related immune regulation. Vitamin D regulates Th1 and Th17 immune responses, which are involved in both autoimmune thyroid diseases (AITD) and uveitis; its deficiency, commonly observed in AITD, may contribute to immune dysfunction and increased susceptibility to non-infectious uveitis [259–261]. Both excess and deficiency of TH exacerbate oxidative stress in endotoxin-induced uveitis, primarily through enhanced generation of ROS (e.g., superoxide anions and hydroxyl radicals) by inflammatory cells. This promotes lipid peroxidation, reflected by elevated malondialdehyde (MDA) levels, and impairs antioxidant defenses, notably by reducing glutathione peroxidase (GPx) activity [262]. This suggests a bidirectional role of TH signaling in uveitis, where excessive or insufficient TH levels may disrupt immune homeostasis and exacerbate inflammatory damage. Future research should focus on elucidating how TH signaling influences immune cell activity, cytokine production, and oxidative stress responses in uveitis.

### TH signaling and TAO

TAO is a complex autoimmune disorder primarily affecting the orbit, commonly occurring in patients with GD or hyperthyroidism [263, 264]. The pathophysiology of TAO is characterized by orbital inflammation, extraocular muscle hypertrophy, adipose tissue expansion, and increased intraorbital pressure, leading to clinical manifestations such as proptosis, diplopia, and, in severe cases, vision-threatening optic neuropathy [265, 266].

Although TAO is commonly associated with hyperthyroidism, it can also occur in individuals with hypothyroidism or normal thyroid function [267]. Thyroid stimulating immunoglobulin (TSI) concentration is associated with disease activity and can serve as a predictor of treatment response to intravenous methylprednisolone in patients with TAO [268]. Other studies have shown that thyroid-stimulating antibodies (TSAbs) are the primary marker of GD and are highly prevalent in TAO patients, demonstrating 100% sensitivity in distinguishing active from inactive and mild from moderate-to-severe TAO [269], with disease activity strongly correlating with circulating TSHR autoantibodies (TSHR-Ab) [270–272]. Additionally, serum 3,3′,5,5′ tetraiodothyroacetic acid (TA4) levels, a product of T4 decarboxylation and oxidative deamination, are elevated in TAO patients [273]. TA4 levels are positively correlated with T4 and negatively correlated with T3 [273]. These antibodies not only serve as independent risk factors for TAO but also predict disease severity and prognosis [274].

TAO is fundamentally driven by a loss of immune tolerance to TSHR [275, 276]. BALB/c mice immunized with the TSHR A-subunit plasmid developed TSHR/insulin-like growth factor 1 receptor (IGF-1R) antibodies, hypothyroidism, orbital inflammation, and proptosis, mimicking TAO [277]. MRI and histopathology confirmed orbital remodeling, while control mice showed no pathology, supporting TSHR as a key driver of TAO [277]. Adoptive transfer of TSHR knockout mouse splenocytes into nude mice induced anti-TSHR autoantibodies, leading to transient hyperthyroidism, followed by hypothyroidism [278]. Importantly, macrophage infiltration in orbital tissues was observed, suggesting that TSHR autoimmunity plays a direct role in TAO-related inflammation [278]. The TAO animal model induced by an adenovirus expressing the TSHR A subunit (Ad-TSHRA) further confirmed this [279].

TSHR, a G protein-coupled receptor (GPCR) with an extracellular A subunit and a membrane-anchored B subunit, serves as a common autoantigen in GD and TAO, exhibiting higher expression in orbital fibroblasts (OFs) from TAO patients [280–285]. Although TSHR is primarily expressed in thyroid cells, its expression in preadipocyte fibroblasts [286] and myofibroblasts [287] in TAO may primarily stem from their bone marrow monocyte progenitor cell origin [286]. TSHR expression in preadipocyte fibroblasts is upregulated by TSH, TSAbs, and IL-6, activating downstream adenylate cyclase/cAMP and PI3K/pAKT pathways, which drive fibroblast-to-adipocyte differentiation and HA production [288–290]. TSHR activation in orbital fibroblasts stimulates cAMP-PKA signaling, upregulating hyaluronan synthase (HAS) 1 and HAS2 to enhance HA production, with its expression correlating with IL-1β levels, linking TSHR signaling to local inflammation [285, 291]. OFs, serving as primary autoimmune targets, play a central role in local immune responses and tissue remodeling [115]. TSHR signaling in OFs induces miR-146a and miR-155, inhibiting ZNRF3 and PTEN to promote fibroblast expansion, while genetic variants of TSHR may enhance its antigenic role in OFs even before adipogenesis, further implicating TSHR in TAO pathogenesis [292, 293].

TSHR and IGF-1R crosstalk plays a crucial role in TAO pathogenesis [294–297]. TSHR and IGF-1R colocalization in OFs enhances synergistic ERK1/2 phosphorylation in TAO [298], leading to the activation of OFs [299]. In vitro studies have demonstrated Graves’ immunoglobulin (Igs) can stimulate HA synthesis in orbital fibroblasts via IGF-1R [300], promoting fibroblast proliferation, ECM remodeling, and cytokine secretion [286]. However, Igs activate IGF-1R indirectly through TSHR/IGF-1R crosstalk after binding to TSHR, rather than by directly interacting with IGF-1R ^323^. FOXO transcription factors act as convergence points for IGF-1R and TSHR signaling in GO, with FOXO1 and FOXO3a repressing excessive fat and HA production in OFs, making them potential targets for nonimmunosuppressive therapy [301–303].

Recent evidence suggests that TH signaling modulates TAO pathogenesis at the epigenetic level. MiR-376b, regulated by T3, influences HAS2 expression and inflammatory cytokine production, indicating that TH-driven microRNA networks contribute to ECM remodeling and inflammatory amplification in TAO [304]. Such findings underscore the role of TH-regulated gene expression in shaping the immune and fibrotic landscape of orbital tissues [304].

**Fig. 6:**
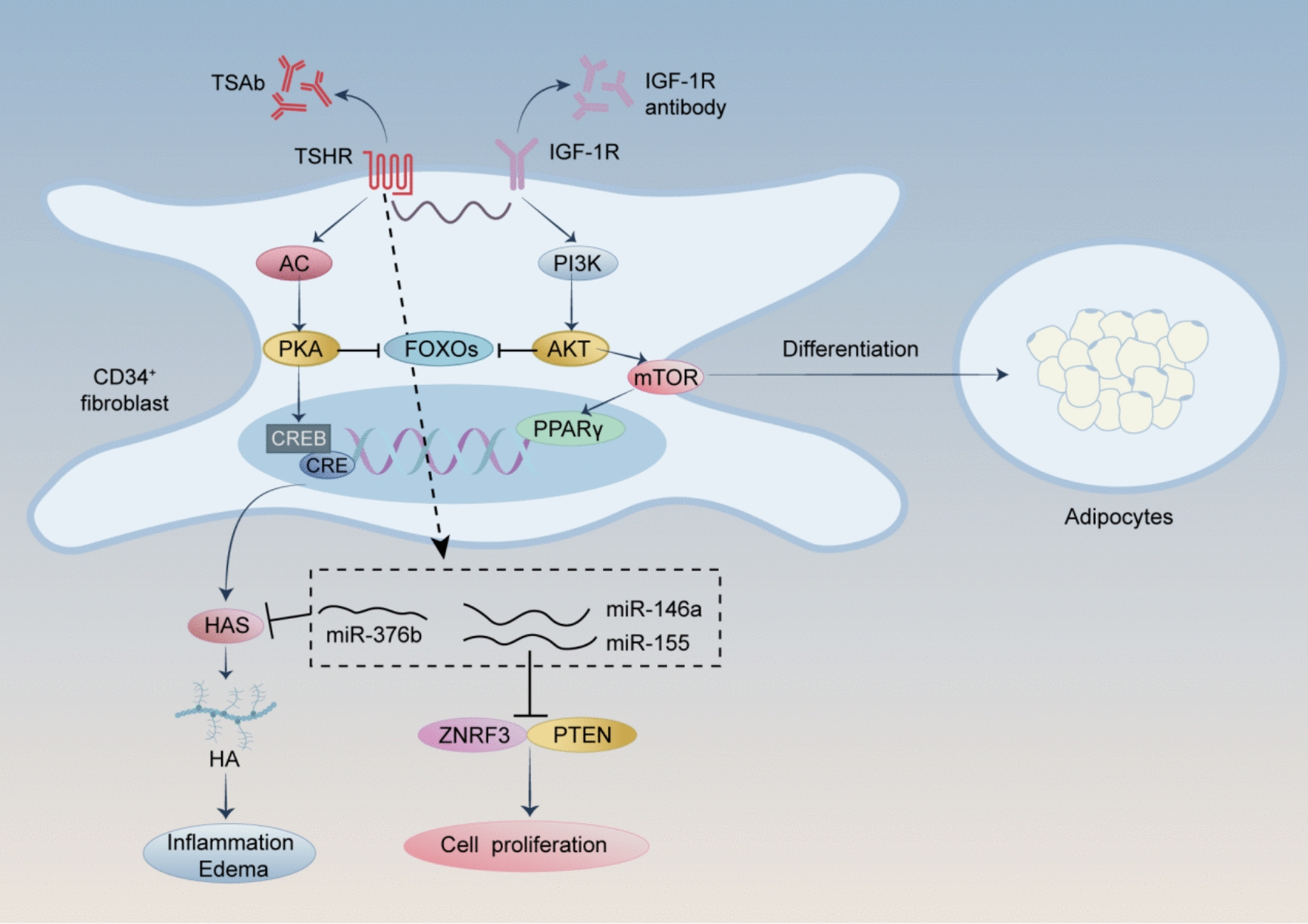
TSHR activates cAMP-PKA and PI3K/AKT pathways to drive fibroblast-to-adipocyte differentiation and HA production. TSHR signaling also upregulates miR-146a and miR-155, which inhibit ZNRF3 and PTEN, promoting fibroblast expansion and tissue remodeling. Additionally, TH signaling modulates TAO via miR-376b, which inhibits HAS2 expression and reduces HA production, contributing to ECM remodeling

The TSHR-IGF-1R signaling axis, coupled with immune activation and epigenetic regulation, constitutes the core mechanistic framework underlying TAO pathogenesis (Fig. 6). This integrated molecular interplay between thyroid autoimmunity, fibroblast activation, and adipogenesis offers critical insights into novel therapeutic strategies, such as TSHR and IGF-1R inhibitors (e. g., teprotumumab) or miRNA-based interventions, aiming to interrupt disease progression and prevent irreversible orbital damage.

## TH signaling and ocular tumors

### TH signaling and retinoblastoma

Retinoblastoma (Rb) is a rare pediatric intraocular malignancy that originates in the developing retina and is driven by biallelic inactivation of the *RB1* gene [305–307]. Children with germline *RB1* mutations are at a significantly higher risk of developing retinoblastoma as well as secondary malignancies later in life [308]. Emerging molecular evidence indicates that TH signaling may be involved in retinoblastoma pathogenesis, particularly through its interaction with TRβ2 and downstream effectors.

The *RB1* gene encodes the retinoblastoma protein (Rb), a nuclear tumor suppressor that regulates cell cycle progression, differentiation, and retinal development. Loss of *RB1* function leads to dysregulated proliferation and tumorigenesis [309]. Retinoblastoma cells exhibit markers of postmitotic cone precursors and rely on cone-specific signaling components, including the TRβ2, for their proliferation and survival [310], suggesting that the TH signaling pathway may increase susceptibility to the oncogenic effects of RB1 mutations [311]. In human retinoblastoma cells, *THRB* is aberrantly transcribed, producing two distinct TRβ2 isoforms: a 54-kDa protein (TRβ2–54), which is predominantly cytoplasmic, and a 46-kDa isoform (TRβ2–46), which is N-terminally truncated, exclusively cytoplasmic, and initiated at Met-79 [312]. The latter, TRβ2–46, functions as an oncogenic TH receptor isoform that drives the expression of kinase-associated protein 2 (SKP2), an E3 ubiquitin ligase that facilitates cell cycle progression, promoting retinoblastoma cell proliferation in an *RB1*-deficient background [312].

Mechanistically, TRβ2 promotes SKP2 expression and simultaneously prevents its degradation via the EMI1/FBXO5 pathway, ensuring sustained SKP2 activity as an E3 ubiquitin ligase that targets key cell cycle inhibitors such as p27 and p130 for degradation [313], thereby driving cell cycle progression and supporting retinoblastoma cell proliferation [314]. TRβ1 has been proposed to function as a tumor suppressor by downregulating EMI1 and SKP2 to inhibit cell cycle progression and prevent aberrant proliferation [314–317]. TRβ2 antagonizes TRβ1, counteracting its suppression of EMI1 and SKP2, thereby promoting uncontrolled proliferation in Rb-deficient retinal cells [314–317]. However, TRβ2 likely exerts additional, yet unidentified, roles in cell cycle regulation and survival, while TRβ1 may have broader tumor-suppressive functions beyond EMI1 and SKP2 regulation [314–317]. Additionally, TRβ2 disrupts an Rb-deficient checkpoint by sustaining EMI1 and SKP2 expression, counteracting the TRβ1-mediated downregulation of SKP2 [318]. Furthermore, TRβ may also function as a tumor suppressor, with its mechanism involving the disruption of Rb and p53 recruitment by the SV40Tag oncoprotein through protein-protein interactions, thereby inhibiting tumor formation [319].

**Fig. 7:**
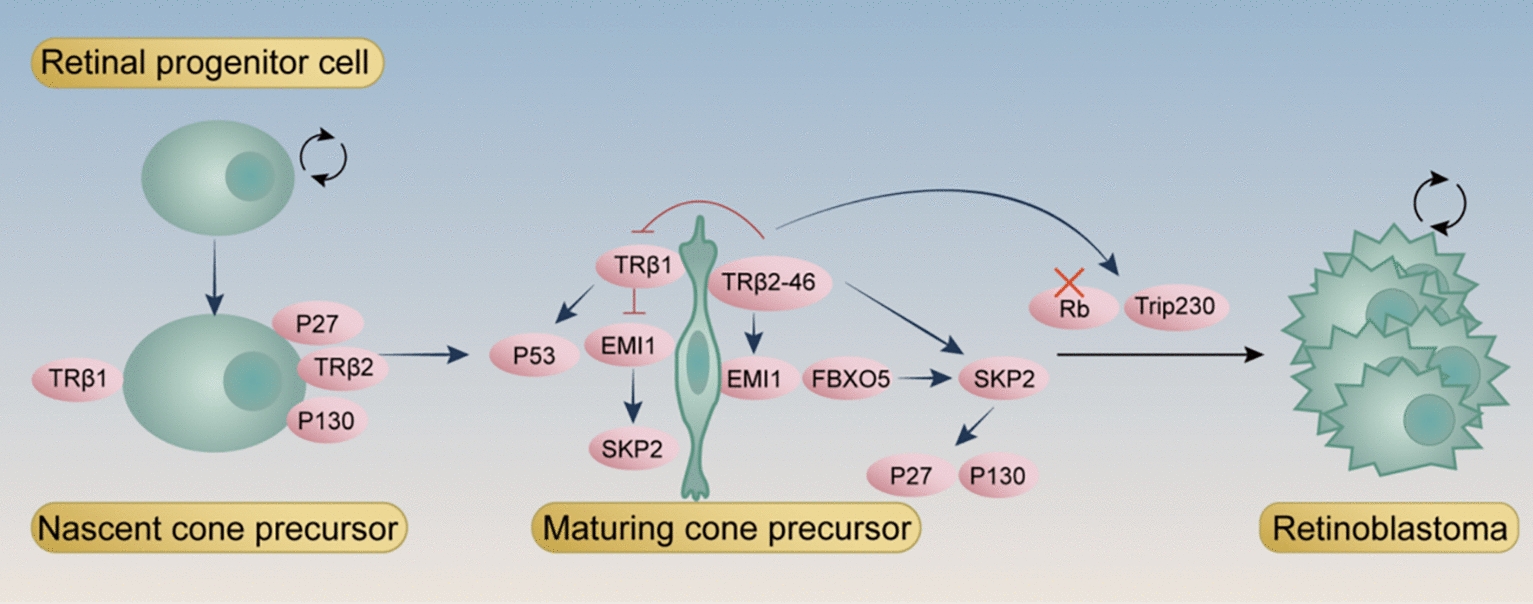
TH signaling plays a key role in retinoblastoma pathogenesis, particularly in RB1-deficient retinal cells. Loss of RB1 function leads to uncontrolled cell proliferation, and TRβ2–46, an oncogenic TH receptor isoform, promotes the expression of SKP2, an E3 ubiquitin ligase, which targets cell cycle inhibitors like p27 and p130 for degradation, driving tumorigenesis. TRβ2–46 counteracts the tumor-suppressive effects of TRβ1, enhancing cell cycle progression in Rb-deficient cells. Normally, Rb sequesters Trip230 to limit TR-mediated transcription. When RB1 is inactivated, this control is lost, allowing TH signaling to drive unchecked gene expression, promoting tumor growth in retinoblastoma

In retinoblastoma, the TH signaling pathway may participate in tumor regulation through its interaction with the Rb protein. Studies have shown that the coactivator Trip230 can simultaneously bind to Rb and form a TH-dependent complex with TRs, enhancing TR-mediated transcriptional activity. Under normal conditions, Rb suppresses excessive TH signaling by sequestering Trip230, thereby inhibiting TR-driven transcription [320]. However, when RB1 is inactivated, this inhibitory control is lost, allowing TR-mediated gene expression to proceed unchecked [320].This mechanism suggests that the TH signaling pathway may influence tumorigenesis in retinoblastoma by regulating cell proliferation and differentiation, closely interacting with the tumor-suppressive functions of Rb protein, and providing new perspectives for studying tumor development and progression [320] (Fig. 7).

### TH signaling and uveal melanoma

Uveal melanoma (UM), also known as choroidal melanoma, is a rare but aggressive intraocular malignancy [321, 322]. As the most common primary intraocular cancer in adults, it has relatively high 5-year survival rates in the localized (85%) and regional (67%) stages [323]. However, the primary challenge in managing UM arises at the metastatic stage, where survival drops significantly to just 16% [323]. Emerging evidence suggests that TH signaling plays a significant role in UM pathogenesis, tumor progression, and potential therapeutic targeting.

Genomic studies have identified frequent chromosomal aberrations in UM, particularly chromosome 3 alterations, which are strongly associated with metastatic potential. Notably, approximately 60% of UM tumors exhibit allelic loss of the *THRB* gene [324]. This loss is common in ciliary body melanomas but also occurs in choroidal melanomas [324]. Some clinical studies have observed altered thyroid hormone profiles in UM patients, raising the possibility of a link between systemic thyroid dysfunction and disease progression. A study comparing UM patients to healthy controls found elevated serum hepatocyte growth factor (HGF) levels alongside reduced TSH levels in UM patients [325]. While HGF is known to drive oncogenic signaling in various cancers, including UM, only serum TSH levels were significantly associated with patient survival, indicating that TH signaling may influence UM progression beyond its genomic alterations [325]. However, the precise mechanisms by which thyroid dysfunction modulates UM tumor behavior remain unclear, warranting further investigation in larger patient cohorts.

Recent findings suggest that TH signaling via the integrin αvβ3 receptor may be a crucial factor in UM progression. This non-genomic TH signaling pathway has been implicated in tumor growth, angiogenesis, and survival in several cancer types. In a preclinical study, the TH-αvβ3 integrin inhibitor tetrac significantly delayed tumor onset in integrin-positive melanoma (B16F10) models, whereas no effect was observed in integrin-negative melanoma cells (B16LS9) [326]. These findings suggest that UM cells may exploit αvβ3-mediated TH signaling to drive tumor progression, presenting integrin-targeted TH modulation as a potential therapeutic strategy.

Animal studies further support the role of TH in UM tumor dynamics. In the B16F10 UM mouse model, induced hypothyroidism (via PTU treatment) significantly delayed tumor growth and extended survival, whereas hyperthyroidism (via T4 administration) accelerated tumor progression and shortened survival [327]. These findings suggest that TH levels actively modulate UM progression, with hyperthyroidism potentially enhancing tumorigenic pathways, whereas hypothyroidism may exert a protective effect. The underlying mechanisms likely involve TH-induced changes in cell proliferation, apoptosis resistance, and angiogenesis, emphasizing the potential of thyroid function modulation as an adjunctive therapeutic approach in UM [328].

Beyond classical TH pathways, TH metabolites have been implicated in tumor regulation. In UM 92. 1 cells, the TH metabolite 3-iodothyronamine (3-T1AM) was found to inhibit tumor growth by activating the TRPM8 ion channel [329]. This activation suppresses VEGF-induced neovascularization while concurrently blocking TRPV1 transactivation, thereby limiting tumor progression. Given that angiogenesis is a critical factor in UM metastasis, 3-T1AM represents a promising therapeutic agent that targets VEGF-driven neovascularization in UM.

## Targeted TH signaling therapy in ophthalmology: emerging molecular strategies

Targeted TH signaling therapy has demonstrated significant success in various medical fields, including pulmonary fibrosis, where the TH analog GC-1 (Sobetirome) exerts antifibrotic effects by activating TRβ, enhancing mitochondrial function, and inhibiting fibroblast activation and ECM deposition [224, 330–332]. Given the broad regulatory role of TH signaling in cellular metabolism, inflammation, and oxidative stress, its therapeutic potential in ophthalmology is increasingly recognized. Modulating TH signaling could provide novel treatment strategies for conditions such as TAO, DED, DR, myopia, glaucoma, UM, AMD, and Rb.

For TAO, TSHR has been identified as a key autoantigen in orbital autoimmunity, making it a primary therapeutic target [263]. The monoclonal antibody K1-70 inhibits TSHR activation and has shown promising clinical trial results, improving thyroid function and TAO symptoms without significant adverse effects [333–336]. In a phase I clinical trial, 18 stable GD patients treated with K1-70 showed improvements in thyroid function as well as in the clinical manifestations of both GD and GO [337]. Different from K1-70, a combination of two TSHR peptides, known as ATX-GD-59, aims to restore immune tolerance by generating regulatory T cells to suppress autoimmune responses against TSHR. Among 10 treated GD patients, seven demonstrated either a complete or partial reduction in free TH levels response to ATX-GD-59 [338]. No severe adverse events were reported. Additionally, TSHR antagonists such as NCGC00229600 (ANTAG2) [339], Org-274179-0 [340], and S37a [334] have shown preclinical potential in modulating TH levels. Beyond TSHR, IGF-1R has been implicated in TAO pathogenesis due to its crosstalk with TSHR [298, 341–344]. Teprotumumab, an anti-IGF-1R monoclonal antibody, has demonstrated efficacy in severe TAO through randomized Phase II and III trials, with significant symptom relief and minimal adverse effects [345, 346]. Linsitinib, an IGF-1R phosphorylation inhibitor, also shows promise in preclinical studies [342, 347].

Elevated TSH levels are linked to the pathogenesis of several ocular diseases, including DED, UM and DR. In thyroid-related DED, TSHR antagonists may potentially protect lacrimal gland function by reducing autoimmune inflammation and preserving tear secretion. In DR, high TSH promotes pericyte loss and microvascular dysfunction, suggesting that TSHR antagonists might offer therapeutic potential for mitigating retinal vascular damage. Conversely, reduced serum TSH levels in UM indicate that TSHR agonists such as Org41841, NCGC00161870, and MS437 may help restore TH homeostasis and suppress tumor progression [348]. However, TSHR-targeting therapies must be carefully designed to avoid systemic disruption of thyroid function.

TRβ-selective agonists have emerged as promising candidates for ocular disease treatment. GC-1 (Sobetirome) and KB2115 (eprotirome) selectively activate TRβ, mimicking T3 while sparing TRα, and have been investigated for conditions such as non-alcoholic fatty liver disease and pulmonary fibrosis. Other TRβ agonists, including resmetirom, ALG-055009, MB07811 (VK-2809), TG68, and HSK31679 [349], show potential for treating DED, DR, and AMD by modulating oxidative stress, apoptosis, and inflammatory signaling in retinal cells. In AMD, TRβ antagonists may protect RPE cells, rods, and cones by modulating cellular stress responses and necroptosis pathways. In Rb, TRβ isoforms exhibit opposing roles, with TRβ2 functioning as an oncogene that promotes SKP2-dependent proliferation, while TRβ1 acts as a tumor suppressor. Targeting TRβ2 could serve as a novel therapeutic approach for Rb by inhibiting its oncogenic functions or restoring TRβ1 activity.

DIOs regulate intracellular T3 availability and have been explored as therapeutic targets in ocular diseases. PTU, a DIO1 inhibitor, is already used clinically for hyperthyroidism [350], while newer inhibitors such as genistein (DIO1-specific) [351] and cefuroxime (DIO2-specific) [352] are in preclinical stages. Cephalosporin Cefuroxime, a novel DIO2 specific inhibitor, selectively inhibits DIO2 activity, alters HPT axis sensitivity, and interferes with the central regulation of TH levels [352]. For DIO3, specific inhibitors have already been developed and are currently in experimental phases for in vitro and in vivo validation [353]. Other well-known inhibitors, such as iopanoic acid, amiodarone, and aurothioglucose, are nonselective and block all three isoenzymes. Resmetirom, a THR agonist, induces *DIO1* expression and enhances mitochondrial function, mimicking T3 activity [354]. In DR, DIO2 agonists or DIO3 inhibitors may potentially increase retinal T3 levels, protecting retinal cells from dysfunction and death by restoring mitochondrial integrity. In AMD, DIO2 inhibitors and DIO3 agonists may improve cone survival and retinal resilience.

TH metabolites also exert significant biological effects, with emerging therapeutic potential in various ocular diseases. In preclinical studies, tetrac, as a physiological metabolite of T4, has been found to compete with TH for binding to the integrin αvβ3 receptor on the plasma membrane, potentially having therapeutic value in DR and UM [179]. A novel derivative, P-bi-TAT, exhibits a more than 400-fold higher binding affinity for integrin αvβ3 compared to Tetrac, demonstrating superior biological activity [355]. Another metabolite, 3,5-T2, generated from T3 deiodination via DIO3 [356], has shown promising metabolic effects, including reducing visceral fat, alleviating hyperglycemia, and improving dyslipidemia in obese hamster models, suggesting its potential application in DR treatment. Additionally, 3,5-T2 can dose-dependently suppress HPT axis activity, leading to a decrease in serum TSH levels, which may provide therapeutic benefits in TAO [357]. 3-T1AM, an endogenous TH derivative, exhibits anti-inflammatory and neuroprotective properties by inhibiting pro-inflammatory cytokine release, reducing microglia-mediated inflammation, and mitigating oxidative stress [358], making it a potential candidate for glaucoma, myopia, DR, AMD, uveitis, and TAO. Moreover, 3-T1AM has been shown to improve lipid metabolism [359], further supporting its potential in DR treatment.

Overall, targeted TH signaling therapy represents a promising frontier in ophthalmology, with potential applications in TAO, DED, DR, and various other ocular diseases (Table 2). While TSHR and IGF-1R modulation offer effective strategies for TAO, TRβ-selective agonists, DIO-targeting and TH metabolite-based therapies provide new opportunities for managing other ocular diseases. Further research is needed to optimize these approaches, ensuring efficacy while minimizing systemic thyroid dysregulation. As our understanding of TH signaling in ocular disease deepens, these targeted therapies may revolutionize the treatment landscape, offering precision medicine solutions for previously untreatable conditions.

**Table 2:** Preclinical and Clinical Agents Modulating the Thyroid Hormone Signaling Pathways in Eye Diseases: Mechanisms, Applications, and Adverse Events

Target	Agent	Clinical/preclinical study	(Potential) treated ocluar disease	(Potential) function in ocular diseases	Used in other fields	Adverse events	References
TSHR antagonists	K1-70	Clinical study	TAO, DED, DR	Stimulate TSHR-Ab and block TSHR activation	GD	Fatigue, lethargy, and diarrhoea	[337]
	ATX-GD-59	Clinical study	TAO, DED, DR	Restore immune tolerance to the TSHR	GD	Headache, nausea, food poisoning, upper respiratory tract, skin, and fungal nail infections	[338]
	NCGC00229600 (ANTAG2)	Preclinical study	TAO, DED, DR	Inhibit basal cAMP, pAKT, and HA production in undifferentiated OFs	/	/	[339]
	Org-274179-0	Preclinical study	TAO, DED, DR	Block cAMP production induced by GD-IgG and M22	/	/	[340]
	S37a	Preclinical study	TAO, DED, DR	Inhibit the TSHR activation and cyclic adenosine monophosphate formation	/	No toxicity of S37a; A remarkable 53% oral bioavailability in mice	[366]
	The novel peptides derived from some cylindrical loops of the leucine-rich repeat domain of the TSHR	Preclinical study	TAO, DED, DR	Reduce serum T4 levels, retro-orbital fibrosis, and tachycardia	/	In immunologically naïve mice, administration of the peptides did not induce any immune response.	[367]
TSHR agonists	Org41841	Preclinical study	UM	Bind to the transmembrane domain of TSHR and luteinizing hormone/chorionic gonadotropin receptor	/	/	[368]
	NCGC00161870	Preclinical study	UM	Increase the levels of mRNAs for TG, TPO, sodium–iodide symporter (NIS), DIO2 and the enzymatic activity of DIO2	Thyroid cancer	/	[369]
	MS437 and MS438	Preclinical study	UM	Engage Gs, Gq, and G12 signaling, resulting in upregulation of thyroid-specific gene expression including TG, NIS, and TSHR	/	/	[370]
	TPY3m	Preclinical study	UM	Upregulate the levels of TG, TPO, and DIO2; Increase the expression of thyroidogenic genes including TG and NIS	/	/	[371]
	D3-βArr (NCGC00379308)	Preclinical study	UM	Selective TSHR activation involved β-arrestin 1 (β-Arr 1)-mediated pathway	Osteoblast differentiation	/	[372]
	MSq1	Preclinical study	UM	Activate protein kinase C (PKC) which in turn suppressed the proliferative signal induced by activation of the predomiant cAMP-PKA pathway of the TSHR	/	/	[373]
IGF-1R antagonists	Teprotumumab	Clinical study	TAO	Block IGF-1R to reduce inflammation and fat accumulation in the orbit	/	Hyperglycemia, an infusion reaction	[346]
	Linsitinib	Preclinical study	TAO	Induce apoptosis and inhibit proliferation of both IGF-1R and TSHR expressing target cells	/	/	[347]
TRβ agonists	GC-1 (sobetirome)	Clinical study	DED, myopia	Promote mitochondrial biogenesis, improved mitochondrial bioenergetics and attenuated mitochondria-regulated apoptosis; Inhibit pathological fibrosis	Pulmonary fibrosis, acute lung injury, and non-alcoholic fatty liver disease, demyelinating disease	/	[330, 374, 375]
	KB-2115 (eprotirome)	Clinical study			Non-alcoholic fatty liver disease	A significant increase in liver enzymes; Cartilage side effects in animals	[374]
	MGL-3196 (resmetirom)	Clinical study			Metabolic dysfunction-associated steatohepatitis (MASH)	Transient mild diarrhoea and nausea	[376]
	HSK31679	Preclinical study				/	[349]
	TG68	Preclinical study			Liver regeneration	/	[377]
	ALG-055009	Preclinical study			Hypercholesterolemia	/	[378]
	MB07811 (VK-2809)	Clinical study			Hypercholesterolemia, non-alcoholic fatty liver disease	/	[379]
DIO1 inhibitor、TPO inhibitor	PTU (propylthiouracil)	Clinical study	TAO, UM	Suppress DIO1 mRNA levels and likely affected the activity of the protein by preventing its lysosomal redistribution	Severe hyperthyroidism, GD	Teratogenic effects	[380] [350]
DIO1 inhibitor	Genestein	Preclinical study	TAO		/	/	[351]
DIO2 inhibitor	Cephalosporin; Cefuroxime	Preclinical study	AMD	Selectively inhibit DIO2, reduce the sensitivity of the HPT axis	/	/	[352]
DIO3 inhibitor	PBENZ-DBRMD; ITYR-DBRMD	Preclinical study	DR	Selectively inhibit DIO3	High-grade serous ovarian cancer (HGSOC)	/	[353]
TH metabolites	Tetrac (3, 5, 3′, 5′⁃tetraiodo⁃ thyroacetic acid)	Preclinical study	UM, DR	Compete with TH in order to bind the plasma membrane integrin αvβ3 receptor that is widely expressed on the surface of vascular endothelial cells in RPE	Non-small cell lung cancer	/	[179]
	P⁃bi⁃TAT	Preclinical study	UM, DR		Pancreatic cancer	/	[355]
	Triac (3, 5, 3′⁃triiodothyroacetic Acid)	Clinical study	Diseases related to cone development	Enter target cells such as cone cells independently of MCT8 and to modulate expression of TH-dependent genes	Allan Herndon Dudley syndrome, thalassemia	No drug-related severe adverse events were reported	[356]
	rT3 (reverse T3)	Preclinical study	/	Enhances dendritic arborization formation in cerebellar Purkinje cells and neurite outgrowth of neurons in an integrin αvβ3 receptor-dependent manner	Human breast cancer and glioblastoma, Allan Herndon Dudley syndrome	/	[381]
	3, 5⁃T2 (3, 5⁃diiodothyronine)	Preclinical study	DR	Dose-dependently inhibit hypothalamic pituitary thyroid axis function	Thyroid cancer, Type 2 diabetes, arhythmia	/	[382]
	3⁃T1AM (3⁃iodothyronamine)	Preclinical study	Glaucoma, myopia, DR, AMD, uveitis, TAO	Inhibit the release of proinflammatory factors, alleviate microglial-mediated inflammatory response and reduce oxidative stress	Obesity, myocardial infarction	/	[358]

## Conclusions and prospects

TH signaling plays a crucial role in regulating glucose, lipid, and protein metabolism, all of which are fundamental to the maintenance of ocular development, structure and function. Disruptions in circulating or intracellular TH levels can lead to significant metabolic imbalances in ocular tissues, contributing to the development or progression of various eye diseases. For example, altered TH levels have been implicated in conditions such as TAO, DR, and AMD. These findings highlight the systemic importance of TH in supporting ocular health and the potential for TH dysregulation to exacerbate metabolic, inflammatory, and fibrotic pathways within the eye.

In preclinical models, TH signaling determines the fate of cone differentiation in human retinal organoids. This has been demonstrated in models where low TH signaling (T3 and TRβ) early specifies the differentiation of S cone, and high TH signaling later promotes the differentiation of L/M cones via temporally dynamic expression of TH-degrading and activating proteins (DIOs) in the retina itself [5]. These processes elucidate the critical role of TH signaling in ocular development and highlight the existence of spatiotemporally specific regulation of DIOs. Supplementation of retinal microvascular endothelial cells with T3 prevents high glucose-induced increases in endothelial nitric oxide synthase, intercellular adhesion molecule 1, and endothelial cell death [189], promoting functional recovery in DR. However, excessive T3 induces photoreceptor degeneration and impairs retinal function via oxidative stress, necroptosis, and inflammation, indicating the bidirectional effects of TH signaling [204]. Given the complex and context-dependent role of TH in ocular disease, further studies are required to elucidate this role.

The expression timing and cellular localization of different TR isoforms in the eye vary significantly, which may underlie the regulation of eye development by TH. This differential expression pattern allows for the development of specifically targeted biologics while minimizing tissue side effects. TRβ-selective thyromimetics are emerging as a novel class of therapeutics with potential applications in ophthalmology. These compounds, designed to mimic the activity of TH in specific tissues, have demonstrated efficacy in non-ocular conditions without adversely affecting other organs such as the heart or bone (TRα-enriched expression) through systemic circulation. While these effects are encouraging, the potential for off-target activity of thyromimetics in tissues with high TRβ expression, such as the retina, needs to be carefully evaluated in the context of ocular disease treatment. In therapeutic applications targeting eye diseases, localized delivery methods, such as eye drops or intravitreal injections, significantly reduce systemic distribution and the associated risk of off-target effects. The high expression of TRβ in the retina may, in fact, enhance the therapeutic efficacy of TRβ-selective thyromimetics by providing a specific target for localized treatment. However, excessive local accumulation of thyromimetics due to improper dosing could theoretically induce"hyperthyroid-like"states in the retina, potentially causing metabolic dysregulation or influencing the progression of preexisting ocular conditions, such as AMD or glaucoma. Therefore, precise control over drug concentration, dosing, and delivery is essential to ensure both safety and efficacy in patients with underlying eye diseases.

Although concerns about the potential for side effects persist, TH signaling represents a promising and emerging target for the treatment of ocular diseases. In recent years, cutting-edge delivery systems like nanoparticles and gene therapy have provided a new idea of localized precision regulation of TH signaling in the context of eye health. By enabling localized precision regulation, these advanced delivery systems can specifically target tissues or cells of interest, such as the retina, while minimizing systemic exposure and adverse effects on non-ocular organs. Nanoparticles can encapsulate TRβ-selective thyromimetics and TH metabolites, allowing controlled and sustained release directly to the eye, reducing the risk of excessive accumulation and off-target activity. Similarly, gene therapy offers the potential to modulate TH signaling pathways at the molecular and cellular level in specific cell types, such as by editing DIOs or TR genes, ensuring precise and localized therapeutic effects. These technologies represent innovative strategies to enhance the efficacy and safety of TH signaling-based treatments for ocular diseases.

The absence of reliable and specific biomarkers in body fluids to monitor TH signaling activity in ocular tissues represents a key limitation in clinical application. Serum markers such as SHBG, used in other fields to reflect TH activity (such as in liver) [12], may lack the precision needed to evaluate ocular-specific effects. Developing biomarkers tailored to assess TH activity in the retina, cornea, sclera, and optic nerve would facilitate more accurate monitoring of therapy and mitigate the risks of overtreatment or adverse effects. Furthermore, the development of assays for measuring circulating thyromimetic levels alongside traditional thyroid function tests is critical to advancing their therapeutic use in ophthalmology. 

Advancing our understanding of TH signaling and the development of TH analogs for ocular diseases holds the potential to revolutionize therapeutic strategies in ophthalmology. By overcoming the limitations of existing diagnostic methodologies and elucidating the precise mechanisms of TH action within ocular tissues, these innovative approaches could pave the way for transformative treatments.

## Data Availability

Not applicable.

## Abbreviations

TH: Thyroid hormone
TRs: Thyroid hormone receptors
DIOs: Deiodinases
TAO: Thyroid-associated ophthalmopathy
DR: Diabetic retinopathy
AMD: Age-related macular degeneration
T3: Triiodothyronine
T4: Thyroxine
HPT: Hypothalamus-pituitary-thyroid
TRH: Thyrotropin-releasing hormone
TSH: Thyroid-stimulating hormone
TBG: Thyroxine-binding globulin
TTR: Transthyretin
HSA: Human serum albumin
FT3: Free fractions of T3
FT4: Free fractions of T4
OATP: Organic anion-transporting polypeptide
MCT: Monocarboxylate transporter
LAT: L-type amino acid transporters
rT3: Reverse T3
T2: Diiodothyronine
TREs: Thyroid hormone response elements
NF-κB: Nuclear factor-κb
HIF-1α: Hypoxia-inducible factor-1α
E: Embryonic day
RPE: Retinal pigment epithelium
P: Postnatal day
RGC: Retinal ganglion cell
SIRT2: Sirtuin 2
cyp27c1: Cytochrome P450 family 27 subfamily C member 1
PTU: Propylthiouracil
TPO: Thyroid peroxidase
KSPG: Keratan sulfate proteoglycan
ECM: Extracellular matrix
ROS: Reactive oxygen species
DED: Dry eye disease
TAO: Thyroid-associated ophthalmopathy
MGD: Meibomian gland dysfunction
GD: Graves’ disease
TSHRs: Thyroid-stimulating hormone receptors
IOP: Intraocular pressure
HA: Hyaluronic acid
ARC: Age-related cataract
DM: Diabetes mellitus
non-PDR: Non-proliferative DR
PDR: Proliferative DR
T2DM: Type 2 diabetes mellitus
T1DM: Type 1 diabetes mellitus
HT: Hashimoto’s thyroiditis
CRP: C-reactive protein
EPO: Erythropoietin
RECs: Retinal endothelial cells
MR: Mendelian randomization
SP: Spherical power
Tg: Thyroglobulin
AL: Axial length
SE: Spherical equivalent
KLF2: Krüppel-like factor 2
CEBPA: CCAAT-enhancer binding protein alpha
TINU: Tubulointerstitial nephritis and uveitis
HTLV-1: Human T-cell leukemia virus type 1
BD: Behcet’s disease
TGA: Thyroglobulin antibodies
AITD: Autoimmune thyroid diseases
TSI: Thyroid stimulating immunoglobulin
TSAbs: Thyroid-stimulating antibodies
TSHR-Ab: TSHR autoantibodies
TA4: Serum 3,3′,5,5′-tetraiodothyroacetic acid
IGF-1R: Insulin-like growth factor 1 receptor
Ad-TSHRA: Adenovirus expressing the TSHR a subunit
GPCR: G protein-coupled receptor
OFs: Orbital fibroblasts
HAS: Hyaluronan synthase
Igs: Immunoglobulin
Rb: Retinoblastoma
SKP2: Kinase-associated protein 2
UM: Uveal melanoma
HGF: Hepatocyte growth factor
3-T1AM: 3-iodothyronamine
